# Evidence of lithium underuse in bipolar disorder: analysis of lithium and antipsychotic consumption, prediction of future trends, regional disparities and indicators of rational and inappropriate use in Europe

**DOI:** 10.1007/s00210-025-04389-0

**Published:** 2025-06-28

**Authors:** Lilly Josephine Bindel, Roland Seifert

**Affiliations:** Institute of Pharmacology, Hannover Medical School, 30625 Hannover, Germany

**Keywords:** Lithium, Antipsychotic, Bipolar disorder, Europe, Pharmaceutical utilisation, Prediction

## Abstract

Mental disorders represent a significant global burden. Despite increasing use of medications, there is no evidence that this burden is decreasing. Lithium has been one of the first-line treatments in bipolar disorder for many years. However, lithium prescribing has plateaued, while antipsychotic use increased over the past decades. This study assesses the current situation and historical trends in 11 European countries for lithium and antipsychotic medications, predicts future trends and presents key characteristics for assessing the appropriate or inappropriate use of lithium. Consumption data for lithium (ATC N05AN) and antipsychotics (ATC N05A) were collected from publicly available sources in European countries, spanning from 1997 to 2024. An analysis of past trends in DID (defined daily dose per 1000 population per day) prescriptions for lithium and antipsychotics was performed and predictions were made until 2030, using ARIMA(0,1,0) models. For each country, the lithium treatment coverage for the prevalence of bipolar disorder was calculated, along with the ratio of antipsychotic to lithium use. Identified similarities and differences were compared between countries and between northern and southern European regions to generalise prescribing patterns. Based on these results, the prescribing behaviour of lithium in the analysed countries was classified as good, moderate or poor. Data were available for 11 countries, including Croatia, Denmark, Estonia, Finland, Germany, Iceland, Italy, the Netherlands, Norway, Spain and Sweden. In all countries, lithium consumption plateaued or declined, while antipsychotic use increased. Projections suggest a continuation of observed trends. Treatment coverage was very low, with the highest rate in Sweden (32.6%) and the lowest in Estonia and Croatia (5.3%). Comparably good prescribing behaviour was observed in the Netherlands, Sweden, Denmark and Iceland. Moderate prescribing was observed in Germany, Spain, Finland and Norway, while poor compliance with lithium prescribing was reported in Italy, Croatia and Estonia. Lithium prescribing is influenced by adherence to guidelines, concerns about side effects and practicability, monitoring infrastructure, clinician training and prescribing preferences. However, antipsychotics also come with severe adverse effects, require extensive monitoring and cause higher healthcare costs compared to lithium. Lithium is increasingly being replaced by antipsychotics in the treatment of bipolar disorder, despite a constant recommendation in guidelines. Lithium remains significantly underused in all countries analysed. A clear north–south shift in prescribing behaviour is observed, with a suggested west–east shift. Northern countries show higher lithium consumption and a lower antipsychotic-to-lithium ratio, reflecting greater adherence to guidelines and more rational prescribing. In contrast, southern countries show lower lithium and higher antipsychotic use. Overall, there is little progress in improving prescribing practices. Withholding lithium from eligible patients is both medically and economically irresponsible. To reduce the burden of mental disorders, effective and evidence-based treatment strategies need to be prioritised, which is currently failed.

**Supplementary Information:**

The online version contains supplementary material available at 10.1007/s00210-025-04389-0.

## Introduction

Mental disorders are ‘among the top ten leading causes of burden worldwide’, with suicide being the 18th leading cause of mortality according to the Global Burden of Disease (GBD) study in 2019 (GBD 2019 Mental Disorders Collaborators 2022). Alarmingly, there is ‘no evidence of global reduction in the burden since 1990’ (GBD 2019 Mental Disorders Collaborators 2022). This stagnation contrasts sharply with the significant increase in pharmaceutical treatments for mental disorders, such as antidepressants and antipsychotics (Ludwig et al 2024).

To explore the reasons for this lack of progress, this study focuses on bipolar disorder, which has a relatively stable global prevalence (GBD 2019; Mental Disorders Collaborators 2022), and lithium, a drug that has been widely acknowledged as the superior first-line treatment due to its efficacy, affordability and long-standing availability (Kessing 2019; Ludwig et al. 2024; Malhi and Bauer 2023; Shorter 2009). Despite its superiority as a mood stabiliser and its well-documented role in suicide prevention in bipolar disorder, lithium use has plateaued in recent decades, while antipsychotic use has risen sharply (Kessing et al. 2016; Ludwig et al. 2024; Airainer and Seifert 2024). It is important to understand the reasons for these discrepancies in prescribing, as they indicate potential irrational prescribing practices that may hinder effective treatment.

Previous research has already identified irrational prescribing behaviour and predicted trends for several drug classes, including antibacterials (Bindel and Seifert 2025a, b) and thyroid hormones (Bindel and Seifert 2025c). This study aims to analyse past and future trends as well as the current state of lithium (ATC N05AN) and antipsychotic (ATC N05A) use (WHO 2024a) in several European countries. While many existing studies focus analysing past trends for single countries, this study provides a comprehensive European overview, identifying key characteristics and prescribing patterns that distinguish rational from irrational prescribing behaviour, and provides an outlook for future outcomes. Our aim is to contribute to effective prevention and treatment of bipolar disorder to reduce the burden of illness on both patients and healthcare systems.

## Methods and materials

### Data collection and preparation

The analysis focuses on the consumption of lithium (ATC N05AN) and its corresponding drug class, antipsychotics (ATC N05A) (WHO 2024a), in all care sectors. All European countries were searched for data on consumption of N05A and N05AN. Consumption data were collected in defined daily doses (DDD) or defined daily doses per 1000 inhabitants per day (DID).

Publicly available data for both N05A and N05AN were available for 11 European countries (Tables S1 and S2). These countries include Croatia (HALMED 2023, 2022, 2018, 2014), Denmark (Sundhedsdatastyrelsen 2024), Estonia (State Medicines Control Agency of Lithuania 2019; Latvian State Agency of Medicines 2016; Estonian State Agency of Medicines 2013), Finland (FIMEA 2021a), Germany (Schwabe and Paffrath 2014, 2015, 2016, 2017; Schwabe et al. 2018, 2019; Schwabe and Ludwig 2020; Ludwig et al. 2021, 2023, 2024), Iceland (Directorate of Health 2025), Italy (Italian Medicines Agency 2024, 2023, 2022, 2021, 2020), the Netherlands (Dutch Healthcare Institute 2024), Norway (Folkehelseinstituttet 2021, 2024), Spain (Ministerio de Sanidad 2025) and Sweden (Socialstyrelsen 2025). The available data cover different time periods from 1997 to 2024, restricting the comparison of changes for the whole available time period between countries. More details on the available periods for each country can be found in Table 1.

**Table 1:** Data availability for the 11 analysed European countries

Country	Covered time period	Data source	Weblink (last accessed: March 03 2025)
Croatia	2019–2022	HALMED 2023, 2022, 2018, 2014	https://www.halmed.hr/en/Promet-proizvodnja-i-inspekcija/Promet/Potrosnja-lijekova/Izvjesca-o-prometu-lijekova/
Denmark	1997–2023	Sundhedsdatastyrelsen 2024	https://www.medstat.dk/en
Estonia	2010–2023	State Medicines Control Agency of Lithuania 2019; Latvian State Agency of Medicines 2016; Estonian State Agency of Medicines 2013	https://statistika.tai.ee/pxweb/en/Andmebaas/Andmebaas__06Ravimistatistika__01Ravimistatistika/ATC-N.px/
Finland	2017–2020	FIMEA 2021a	http://raportit.nam.fi/raportit/kulutus/laakekulutus_e.htm
Germany	2013–2022	Schwabe and Paffrath 2014, 2015, 2016, 2017; Schwabe et al. 2018, 2019; Schwabe and Ludwig 2020; Ludwig et al. 2021, 2023, 2024	-
Iceland	2015–2024	Directorate of Health 2025	https://app.powerbi.com/view?r=eyJrIjoiZmRiMGJkNmMtZWQ4NC00NmUzLTlkY2UtZTQ0NDk5ZjZmMDE2IiwidCI6Ijc2NGEzMDZkLTBhNjgtNDVhZC05ZjA3LTZmMTgwNDQ0N2NkNCIsImMiOjh9
Italy	2014–2023	Italian Medicines Agency 2024, 2023, 2022, 2021, 2020	https://www.aifa.gov.it/en/uso-dei-farmaci-in-italia
Netherlands	2019–2023	Dutch Healthcare Institute 2024	https://www.gipdatabank.nl/
Norway	2004–2020	Folkehelseinstituttet 2021, 2024	https://www.norpd.no/; https://www.fhi.no/he/legemiddelregisteret/ledemiddelstatistikk/
Spain	2010–2023	Ministerio de Sanidad 2025	https://www.sanidad.gob.es/areas/farmacia/consumoMedicamentos/ATC/home.htm
Sweden	2006–2023	Socialstyrelsen 2025	https://sdb.socialstyrelsen.se/if_lak/val_eng.aspx

Data were preferably collected in DID prescriptions. Where only DDD prescriptions were available, population size data were used to calculate DID prescriptions. This was necessary for Germany and Sweden. While Sweden provided population data in its drug statistics for 2006–2023 (Socialstyrelsen 2025), population data for Germany were available for 2013–2023 (Eurostat 2025).

DID prescriptions were calculated using the following formula:

$$\text{DID prescriptions}=\frac{\text{DDD prescriptions}}{\text{population}\times 365}\times 1000$$

### Calculation of treatment coverage and ratio between antipsychotics and lithium

Lithium treatment coverage for bipolar disorder (Table S3) was calculated to relate lithium use to the prevalence of bipolar disorder in Europe (IHME 2024). This measure assesses the percentage of people with bipolar disorder receiving lithium treatment. It is therefore necessary to multiply the prevalence by a factor of 10, as the % is expressed per 100, whereas DID prescriptions are expressed per 1000 inhabitants. To derive the treatment coverage, the DID prescriptions are divided by the transformed prevalence. Coverage below 100% indicates underuse, while coverage above 100% indicates potential overtreatment.

The treatment coverage was calculated as follows:

$$\mathrm{Treatment}\;\mathrm{coverage}\;\mathrm{in}\;\%=\frac{\mathrm{DID}\;\mathrm{prescriptions}}{\mathrm{prevalence}\;\mathrm{in}\;\%\;\times100}$$

The ratio of antipsychotics to lithium (Table S4) was used to represent the relationship between antipsychotic and lithium use, indicating how much more frequently antipsychotics are used than lithium. A higher ratio reflects greater reliance on antipsychotics than on lithium.

The ratio was calculated as follows:

$$\text{ratio}= \frac{\text{DID prescriptions of antipsychotics }\left(\text{N}05\text{A}\right)-\text{DID prescriptions of lithium }(\text{N}05\text{AN})}{\text{DID prescriptions of lithium }(\text{N}05\text{AN})}$$

DDD costs are calculated for the most recent year to assess a potential influencing factor. Where only total costs were available, DDD costs were calculated using DID prescriptions and the population size of the country (Eurostat 2025). The factor 365/1000 is needed to convert the unit DID into DDD. If total expenditure is given in foreign currency, it is converted into euro using the corresponding conversion factor (ECB 2022; finanzen.net 2025).

The DDD costs were calculated as follows:

### Forecast of consumption trends with ARIMA models

The autoregressive integrated moving average (ARIMA) model was chosen for its ability to capture different components of a time series, including autoregressive (AR) behaviour, differentiation (I) and moving average (MA) components. The model is defined by its three parameters ARIMA(*p, d, q*). The autoregressive term (*p*) represents the number of past observations that influence the current forecast, with higher values increasing the reliance on historical data but potentially adding complexity. The differencing order (*d*) ensures stationarity by eliminating trends, although excessive differencing can lead to overfitting and loss of meaningful structure. The moving average term (*q*) determines how past errors affect the current forecast. This model is particularly well suited to data sets where past values significantly influence future trends (Bindel and Seifert 2025a, b, c).

Several Python libraries were used to identify the optimal ARIMA parameters (Table S5), including ‘pandas’ (McKinney 2010), ‘pmdarima’ (pypi 2024), ‘statsmodels’ (Seabold and Perktold 2010) and ‘openpyxl’ (pypi 2024). The analysis was carried out in Google Colab (https://colab.research.google.com/). The ‘pandas’ library facilitated the handling and processing of tabular data, particularly for loading and editing Excel files. The ‘pmdarima’ library provided the ‘auto_arima’ function, which automates the selection of ARIMA parameters based on the BIC (Bayesian Information Criterion). The ‘statsmodels’ library, specifically the ‘adfuller’ function, was used to perform the Augmented Dickey-Fuller (ADF) test, which assesses the stationarity of each time series (Dickey and Fuller 1979).

After testing several approaches, the global ARIMA(0,1,0) model was found to be the most appropriate. This was further supported by the patterns observed in the autocorrelation function (ACF) and partial autocorrelation function (PACF) plots, which indicated the need for first-order differencing, while showing no significant patterns in the differenced series. ARIMA models were developed using SPSS, without outlier detection. Long-term forecasts were made up to 2030, providing consumption trends for all countries (Tables S6 and S7). Forecasting models were successfully implemented for lithium and antipsychotic consumption in almost all countries, except for lithium in Croatia, where insufficient data failed to develop a model.

To assess model reliability (Table S8), the assessment focused on fit metrics, including *R*-squared, mean absolute percentage error (MAPE) and maximum absolute percentage error (MaxAPE), as well as the range of confidence intervals, expressed as the relative range of the upper confidence limit (UCL) and lower confidence limit (LCL). A good model fit is characterised by a stationary *R*-squared above 0.65, *R*-squared above 0.85, MAPE below 6 and MaxAPE below 15. Moderate fit corresponds to *R*-squared between 0.4 and 0.65, *R*-squared between 0.6 and 0.85, MAPE between 7 and 20 and MaxAPE between 16 and 40. Poor fit is indicated by a steady-state *R*-squared below 0.4, *R*-squared below 0.6, MAPE above 20 and MaxAPE above 40. Taken together, these metrics provide a comprehensive assessment of ARIMA model performance, allowing a balanced evaluation of forecasts across countries and time series, categorising them as good, moderate or poor (Bindel and Seifert 2025a, b, c).

### Evaluation of data and categorisation of prescribing behaviour of lithium for bipolar disorder

To classify the prescribing behaviour of the countries analysed, patterns were identified and categorised into three groups: good, moderate and poor. The categorisation is based on the average of all countries included and valid in comparison.

Good prescribing practice was defined as lithium DID prescriptions above 1.0 DID, lithium treatment coverage above 22% and a ratio below 12. Moderate prescribing was defined as lithium DID prescriptions between 0.5 and 1.0 DID, treatment coverage between 10 and 22% and a ratio between 12 and 25. Poor prescribing is characterised by lithium DID prescriptions of less than 0.5 DID, treatment coverage of less than 10% and a ratio of more than 25. For DID prescriptions of antipsychotics, less than 12 DID is defined as low, between 12 and 20 DID as average and more than 20 DID as high.

**Fig. 1:**
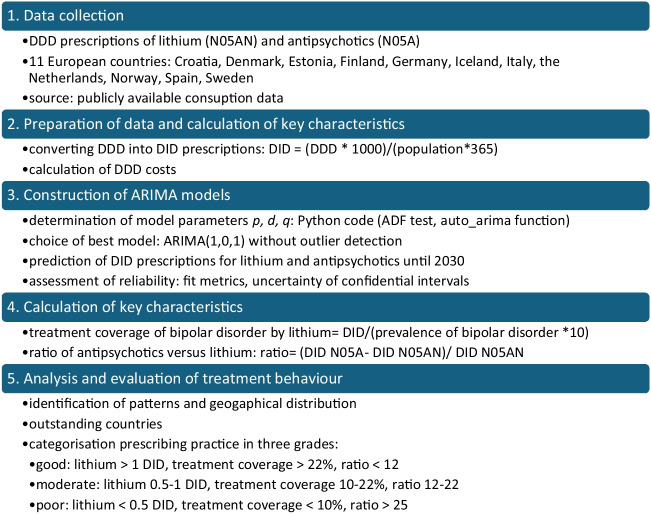
Methodological approach

Microsoft Excel and SPSS were used for calculations, data analysis and visualisation. The methodological procedure is illustrated in Fig. 1.

## Results and discussion

### Development of DID prescriptions for lithium

**Fig. 2:**
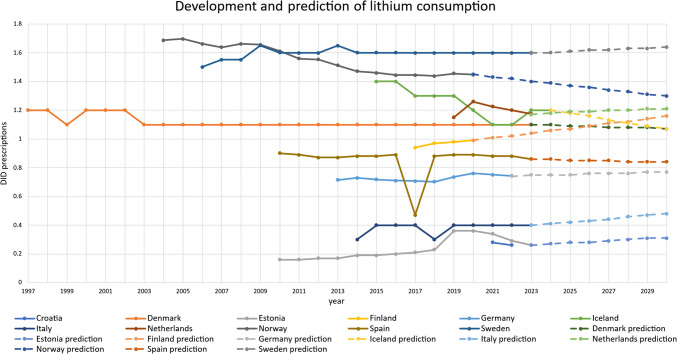
Past development and prediction of DID prescriptions for lithium (N05AN) in the analysed countries

Trends in lithium consumption in the 11 analysed countries were assessed on the basis of the defined daily dose per 1000 inhabitants per day (DID) within the available years (Fig. 2 and Table S1). The following analysis examines the overall trend over the available observation period, changes in the most recent years since 2020 and differences in consumption levels between countries. Furthermore, possible reasons for the observed changes in the trajectories are discussed.

Comparing the development for the first and the last data point for each individual country, an increase is reported for Croatia (between 2019 and 2022; not quantifiable, see Table S1), Estonia (+ 62.5% relative change between 2010 and 2023), Finland (+ 5.3% between 2017 and 2020), Germany (+ 4.1% between 2013 and 2022), Italy (+ 33.3% between 2014 and 2023), the Netherlands (+ 2.0% between 2019 and 2023) and Sweden (+ 6.5% between 2006 and 2023) (Table 2). Decreases were reported for Denmark (− 8.3% between 1997 and 2023), Iceland (− 14.3% between 2015 and 2024), Norway (− 14.3% between 2004 and 2020) and Spain (− 4.4% between 2010 and 2023). Importantly, compared time periods differ, restricting the comparison of changes between countries.

**Table 2:**
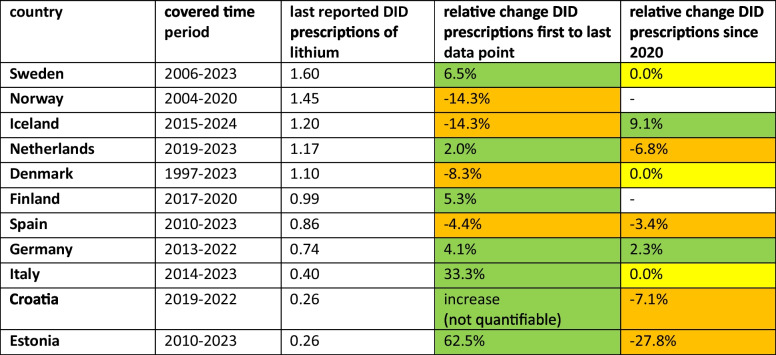
Consumption and trends for lithium (ATC N05AN) for the countries analysed. The available period, the most recent reported consumption in DID prescriptions and the relative changes for the whole period and since 2020 are shown. Increases are green-coloured, plateauing is yellow-coloured and decreases are orange-coloured. Countries are listed in descending order of its most recent consumption

In recent years since 2020, DID prescriptions increased in Germany (+ 2.3% relative change) and Iceland (+ 9.1%), while they decreased in Croatia (− 7.1%), Estonia (− 27.8%), the Netherlands (− 6.8%) and Spain (− 3.4%). No change was reported for Denmark, Italy and Sweden. An assessment is not possible for Finland and Norway because the latest data found is for 2020. Overall, most countries have plateaued in their trends, including Sweden, Denmark, Spain, Germany and Italy. A decreasing development is depicted for Norway, Iceland, the Netherlands and Estonia, while an increase might take place in Finland. Limited assessments include Croatia, Finland and the Netherlands due to a short period of available data.

The most recently reported DID prescriptions vary considerably between the countries analysed. Croatia and Denmark have the lowest DID (0.26), followed by Italy (0.4 DID), Germany (0.74 DID) and Spain (0.86 DID). In the middle are Denmark (1.1 DID), Iceland (1.2 DID) and Norway (1.45 DID). Sweden has the highest number of prescriptions with 1.6 DID.

While many countries show stable use over the period analysed, there are changes in some time spans. Significant decreases are observed for Norway between 2010 and 2016 and for Iceland in 2017, while a decreasing trend is observed in Germany.

Sudden peaks are observed for Spain in 2017 and Italy in 2018. In addition, strong decreases are reported for Estonia and the Netherlands from 2021 to 2023. Declining trends were confirmed and recognised in other regions (Shuy et al. 2024; Kessing et al. 2016; Karanti et al. 2016; Bohlken et al. 2020). Although lithium has been confirmed to be effective in bipolar disorder and in preventing suicide (Cipriani et al. 2013; Schwabe et al. 2018), it has side effects such as renal toxicity (McKnight et al. 2012) and parathyroid dysfunction (Shine et al. 2015), and a narrow therapeutic index (McKnight et al. 2012; Shine et al. 2015; Gitlin 2016; Schwabe et al. 2018) that requires monitoring through frequent blood tests (Gitlin 2016). There is evidence that lithium has been replaced by other agents for treatment, such as antipsychotics (Kessing et al. 2016; Bohlken et al. 2020; Schwabe et al. 2019). The reported decrease in prescriptions in recent years may be due to reduced access to health services and monitoring challenges during the COVID pandemic. Notable increases were reported in the late 2010 s, particularly in Estonia, Germany, Finland and Croatia. This may be due to the explicit recommendation of lithium as a first-line treatment for bipolar disorder in guidelines (Ludwig et al. 2024; Malhi et al. 2017; Gitlin and Bauer 2024), as well as the recognition of a potential neuroprotective effect (Puglisi-Allegra et al. 2021; Rybakowski et al. 2018).

The overall trend has reached a plateau in most countries, including Croatia, Denmark, Finland, Germany, Italy, the Netherlands, Norway, Spain and Sweden, while a long-term decrease is observed in Estonia and Iceland. The number of DID prescriptions varies widely, but there is an overall plateauing or decreasing consumption of lithium. Divergent consumption volumes or trends between countries cannot be explained by events or changes in guidelines, suggesting country-specific or cultural differences in prescribing patterns.

### Prevalence of bipolar disorders

The prevalence of bipolar disorder is essential to evaluate lithium use and to calculate treatment coverage. Therefore, an assessment of the prevalence of bipolar disorder in Europe is needed, along with further insights into global prevalences and epidemiological characteristics.

According to data from the Global Burden of Disease (GBD) study, the prevalence of bipolar disorder in 2021 is 0.56% in Central Europe, Eastern Europe and Central Asia, while the global prevalence is slightly lower at 0.49% (IHME 2024). Looking at trends since 1990, there has been little variation, with the overall rate remaining relatively stable. In Europe and Central Asia, prevalence increased slightly from 0.52% in 1990 to 0.57% in 2006, but then declined to 0.56% by 2021 (Table S3). Globally, there is a slight increase from 0.44% in 1990 to 0.49% in 2021. Furthermore, no significant differences in prevalence by sex were observed, and differences between countries in the European-Central-Asian region remain minimal (GBD 2019 Mental Disorders Collaborators 2022). In general, there has been no significant change in the prevalence of bipolar disorder or mental disorders overall (GBD 2019 Mental Disorders Collaborators 2022; Merikangas et al. 2011).

Various demographic, genetic and environmental risk factors contribute to the development of bipolar disorder (Rowland and Marwaha 2018). Pathophysiological mechanisms like immune-inflammatory changes can destabilise neurotransmitter signalling, resulting in structural brain abnormalities and functional brain alterations (Magioncalda and Martino 2022). Differences in prevalence between ethnic groups remain unclear, with some studies reporting variation and others not (Rowland and Marwaha 2018). However, a genetic contribution has been consistently identified and confirmed (Escamilla and Zavala 2008; O’Connell and Coombes 2021; Gurung and Prata 2015). Other risk factors include psychological stressors, substance misuse and lifestyle (Rowland and Marwaha 2018; Zhao et al. 2016).

The slightly higher prevalence of bipolar disorder in Europe and Central Asia compared with the global average may be explained by several factors. Given the strong genetic component, it is possible that a higher proportion of individuals in these regions carry high-risk genetic variants (Kerner 2014). In addition, substance abuse, particularly alcohol and cannabis use, is more prevalent in Europe than the global average (Castaldelli-Maia and Bhugra 2022), as is obesity (WHO 2022, 2024b). However, significant discrepancies in reported prevalence rates between studies may be due to differences in study methodology, including differences in diagnostic tools used to identify bipolar disorder (Caetano et al. 2013) or cultural differences in symptom recognition (Merikangas et al. 2011; Li et al. 2023).

In summary, the prevalence of bipolar disorder has remained relatively stable and consistent across different regions. However, variations in reported prevalence rates may lead to discrepancies in treatment coverage calculations when compared with other publications. ‘Differences in prevalence rates between countries may reflect methodological differences in diagnostic procedures or assessment methods as well as true differences in disease prevalence’ (Merikangas et al. 2011).

### Treatment coverage of bipolar disorders with lithium

Lithium prescription rates need to be related to prevalence in order to assess the sufficiency of lithium treatment of bipolar disorder, to assess prescribing behaviour and to identify patterns. As lithium is the superior first-line treatment for bipolar disorder, every patient with bipolar disorder should be considered for treatment with lithium (Kessing 2019; Ludwig et al. 2024; Malhi and Bauer 2023). Furthermore, lithium is indicated for severe depression and schizoaffective mixed psychosis (Rote Liste 2025). However, it was decided to only include the prevalence of bipolar disorder in the treatment coverage calculation, which could lead to an overestimation of perceived values. To calculate treatment coverage, DID prescriptions are divided by the expected number of patients per 1000 inhabitants (calculated by multiplying the prevalence by 10). The most recent available prescribing data and changes in coverage over the period analysed are analysed below, followed by a comparison with the available literature.

**Fig. 3:**
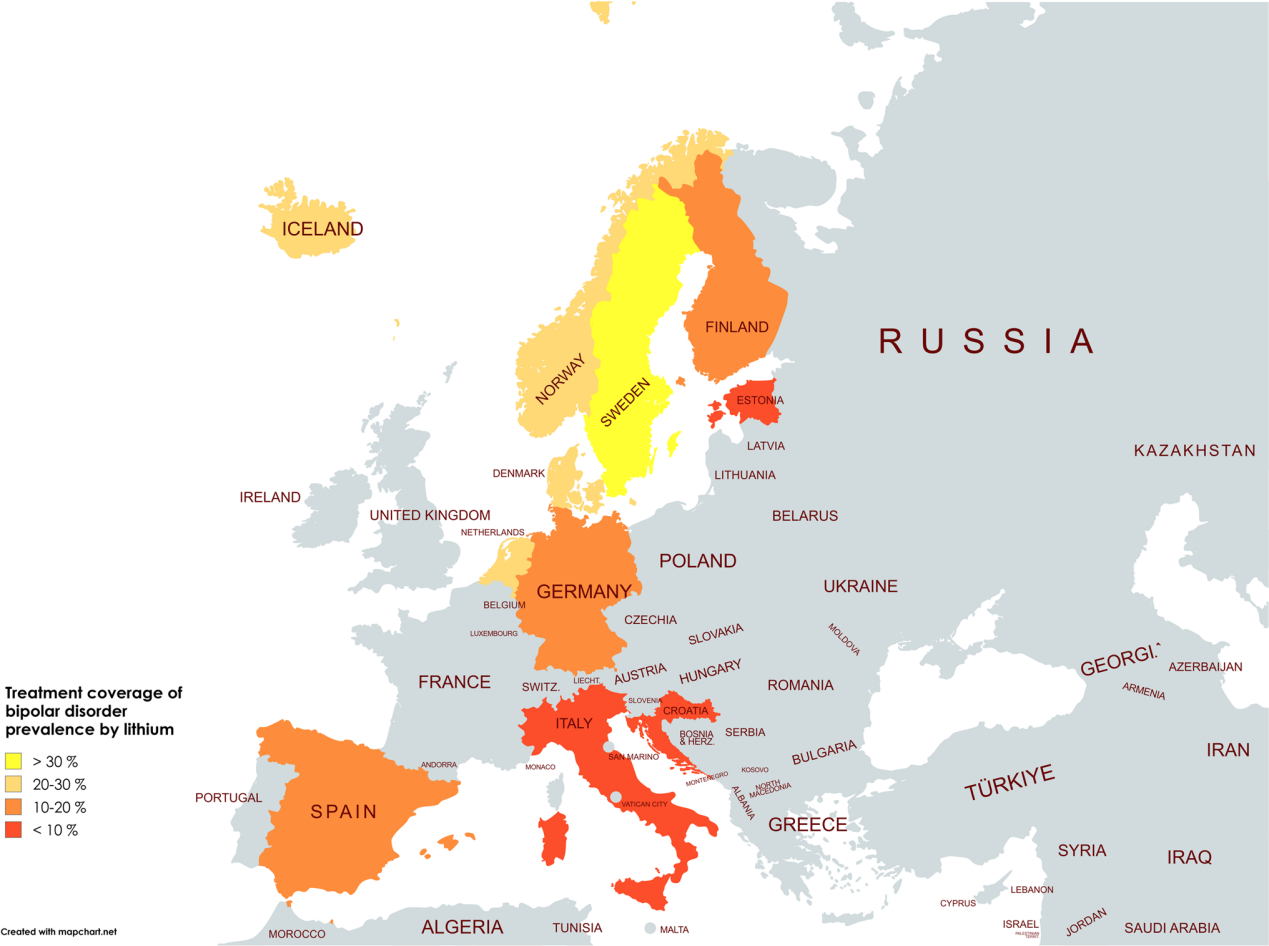
Lithium treatment coverage for bipolar disorder prevalence in the countries analysed for the most recent consumption data. Yellow countries are those with coverage above 30%, beige countries have 20–30% coverage, orange countries have coverage between 10 and 20% and red countries have coverage below 10%. Grey indicates countries for which no data were available. The map was created with mapchart.net (https://www.mapchart.net/index.html)

Sweden has the highest treatment rate (32.6%), followed by Norway (29.5%), Iceland (24.5%), the Netherlands (24.0%), Denmark (22.4%) and Finland (20.2%). Below the average (18.6%) are Spain (17.6%), Germany (15.2%), Italy (8.2%), Estonia (5.3%) and Croatia (5.3%) (Table S3 and Fig. 3). A slight upward trend can be observed in Croatia, Estonia and Finland, while Germany, Italy, the Netherlands and Sweden have reached a plateau after an initial increase. On the other hand, Denmark, Iceland, Norway and Spain recorded a decrease. It is worth noting that when a decline occurs, it tends to be more substantial than the increases observed.

Comparisons with the literature show that treatment coverage is often assessed based on diagnosed patients rather than overall prevalence. However, this has the limitation that the perceived ratios only include diagnosed patients. This means that a significant proportion of undiagnosed individuals who also need to be accounted for are potentially overlooked, and treatment coverage is overestimated. Reported coverage rates among diagnosed bipolar patients include 70.0% in the Netherlands (Renes et al. 2018), 52.4% in Sweden (Sköld et al. 2021), 37.0% in Finland (Karanti et al. 2015), 34.0% in Denmark (Kessing et al. 2016), 26.2% in Germany (Bohlken et al. 2020) and 10.4% in Spain (Grande et al. 2013). Across Europe, general coverage is reported to be 35.7% (Shuy et al. 2024). Although there are differences between coverage rates based on prevalence and those based on diagnosed patients, the overall pattern recognised in coverage of prevalence and DID prescriptions of lithium remains consistent. Countries with high prevalence-based coverage also have high lithium treatment rates among diagnosed patients, whereas countries with lower prevalence-based coverage tend to have lower treatment rates among diagnosed patients.

Overall, lithium treatment coverage remains worryingly low at less than one-third of the prevalence (Fig. 3). Within the countries analysed, coverage varies considerably, ranging from 32.6% to 5.3%. There is little variation in trends over time. This suggests an overall stagnating situation, which is confirmed by several studies (Ludwig et al. 2024; Airainer and Seifert 2024). Despite strong guideline recommendations (Kessing 2019; Ludwig et al. 2024), barriers to lithium treatment persist, including a high awareness about its narrow therapeutic index, long-term side effects and limited scientific and financial interest (Ludwig et al. 2024; Airainer and Seifert 2024). Importantly, antipsychotics come with severe adverse effects as well and also require extensive monitoring (Ludwig et al. 2025; Azfr Ali et al. 2021). While literature-based treatment coverage figures differ because they focus on diagnosed patients rather than prevalence, a rational approach to the management of bipolar disorder requires not only guideline-adherent treatment, but also efforts to identify undiagnosed cases to improve overall treatment rates.

### Development in prescriptions of antipsychotics and comparison with consumption of lithium

Lithium (ATC N05AN) belongs to the broader class of antipsychotics (ATC N05A). While lithium is primarily indicated for bipolar disorder, acute mania and suicidality (Ludwig et al 2024), antipsychotics are indicated and used for a wider range of conditions, including schizophrenia, bipolar disorder and ‘off-label’ uses such as sleep disorders or unipolar depression (Ludwig et al 2024). To contextualise trends in lithium prescribing, it is useful to examine trends in the drug class, particularly as other substances in this class are also used to treat bipolar disorder. The following analysis examines the development of DID prescriptions of antipsychotics (Table S2), compares their trends with lithium and assesses their relationship. In addition, the findings are validated by a literature review and possible reasons for the observed trends are discussed.

Antipsychotic use increased in all 11 European countries analysed (Table 3). Although the relative changes are not fully comparable due to the different time periods, they provide an indication of the dynamics of the trend within each country. The largest increase is observed in Croatia (+ 100.4% between 2007 and 2022) and the smallest in the Netherlands (+ 1.1% between 2019 and 2023). Significant increases are recorded in Estonia (+ 77.0% between 2010 and 2023), Denmark (+ 49.5% between 1997 and 2023), Spain (+ 33.4% between 2010 and 2023) and Italy (+ 26.2% between 2014 and 2023). More moderate increases are observed in Sweden (+ 15.9% between 2006 and 2023), Iceland (+ 14.8% between 2015 and 2023), Germany (+ 13.2% between 2013 and 2022), Norway (+ 9.9% between 2004 and 2020) and Finland (+ 7.5% between 2017 and 2020). The latest reported consumption of antipsychotics ranges from 24.0 DID in Norway to 8.2 DID in the Netherlands. Relatively high consumption is found in Finland (22.2 DID), Croatia (16.1) and Spain (14.5), while below-average levels (14.2 DID) are reported by Iceland (14.0 DID), Denmark (13.6), Germany (12.4), Estonia (10.8), Italy (10.6) and Sweden (9.9).

**Table 3:** Consumption and trends for antipsychotics (ATC N05A) for the countries analysed. The available period, the most recent reported consumption in DID prescriptions and the relative changes for the whole period are depicted. Countries are listed in descending order of its most recent consumption

Country	Covered time period	Last reported DID prescriptions of antipsychotics	Relative change DID prescriptions first to last data point
Norway	2004–2020	24.03	9.9%
Finland	2017–2020	22.15	7.5%
Croatia	2007–2022	16.05	100.4%
Spain	2010–2023	14.50	33.4%
Iceland	2015–2023	14.00	14.8%
Denmark	1997–2023	13.60	49.5%
Germany	2013–2022	12.37	13.2%
Estonia	2010–2023	10.80	77.0%
Italy	2014–2023	10.60	26.2%
Sweden	2006–2023	9.85	15.9%
Netherlands	2019–2023	8.19	1.1%

To better understand the relationship between lithium and antipsychotic use, the ratio of antipsychotics excluding lithium to lithium itself is calculated (ratio = (N05A-N05AN)/N05AN). The most recently reported ratio gives an indication of the balance between antipsychotic and lithium treatment, while the trend over time shows changes (Table S4). The latest ratio varies from 5.2 in Sweden to 60.7 in Croatia, indicating, for example, that in Sweden, antipsychotics are used 5.2 times more often than lithium. High ratios are reported in Estonia (40.5), Italy (25.5) and Finland (21.4), while moderate ratios are found in Norway (16.3), Spain (15.9), Germany (15.6) and Denmark (11.4). Lower ratios are found in Iceland (10.7) and the Netherlands (6.0). In almost all countries, the ratio is increasing, except in Italy, where it is decreasing (from 27.0 in 2014 to 25.5 in 2023) and in the Netherlands, where it remains stable (6.0 from 2019 to 2023). All other countries show an increase, such as Croatia (from 55.4 in 2021 to 60.7 in 2022), Denmark (from 6.6 in 1997 to 11.4 in 2023), Estonia (from 37.1 in 2010 to 40.5 in 2023), Finland (from 20.9 in 2017 to 21. 4 in 2020), Germany (from 14.4 in 2012 to 15.6 in 2021), Iceland (from 7.7 in 2015 to 10.7 in 2024), Norway (from 12.5 in 2004 to 16.3 in 2020), Spain (from 11.1 in 2010 to 15.9 in 2023) and Sweden (from 4.7 in 2006 to 5.2 in 2023).

This indicates a significant increase in the use of antipsychotics compared with lithium, suggesting that other antipsychotics are prescribed more frequently than lithium. A low ratio indicates a more rational use of lithium, with lithium being chosen more frequently as a treatment, whereas a high and increasing ratio may indicate that lithium is underused and being replaced by other antipsychotics. However, interpretation remains complex because antipsychotics are used for a wide range of indications (Ludwig et al. 2024) and are often used in combination with each other (Ortiz-Orendain et al. 2017; WHO 2012).

It is striking that the use of antipsychotics has increased significantly in all countries, while lithium prescriptions have largely plateaued or decreased. The literature confirms these observed trends, with antipsychotic use increasing and lithium prescriptions decreasing (Hálfdánarson et al. 2017; Hojlund et al. 2019; Shuy et al. 2024). A notable factor is the significant increase in the use of atypical antipsychotics (Hálfdánarson et al. 2017; Ludwig et al. 2024), possibly due to their perception as more effective and with fewer side effects (Ucok and Gaebel 2008), which is not the case due to other and unknown similar severe adverse effects (Stroup and Gray 2018; Ludwig et al. 2025), as well as extensive marketing efforts to promote the use of antipsychotics (Apollonio 2022). Expanded indications and a high rate of off-label use, around 40–75%, could the main reasons for the strong increase in antipsychotic prescriptions (Hálfdánarson et al. 2017). There is strong evidence that lithium is gradually being replaced by antipsychotics (Greil et al. 2025; Lin et al. 2020; Jauhar and Young 2019). Variations in use between countries reflect differences in licencing and prescribing policies, including approved indications, access to psychiatrists and physicians, cultural attitudes, pricing, reimbursement policies and availability of antipsychotics (Hálfdánarson et al. 2017; Cookson 2001).

In summary, the large increase in antipsychotic prescriptions, the decrease in lithium usage and the increasing ratio of antipsychotics to lithium suggest an ongoing shift from lithium to antipsychotic treatment. This trend is worrying because lithium has always been the superior treatment for bipolar disorder (Lin et al. 2020; Kessing 2019; Ludwig et al. 2024; Malhi and Bauer 2023). The use of less effective treatments can have serious consequences, particularly for suicide prevention and severe mental disorders, along with higher healthcare costs. Urgent action is therefore needed to reverse this trend and ensure that lithium remains a central component of bipolar disorder treatment (Malhi et al. 2023).

### Prediction of trends for lithium and antipsychotics until 2030

**Fig. 4:**
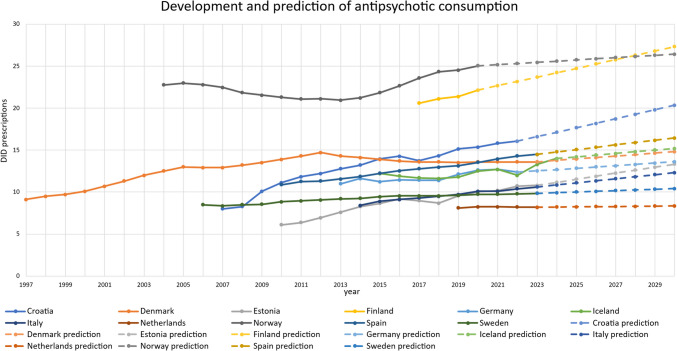
Past development and prediction of DID prescriptions for antipsychotics (N05A) in analysed countries

The ARIMA(0,1,0) model was used to project DID prescriptions of lithium and antipsychotics for the years following the latest reported data up to 2030 (Figs. 2 and 4). The main results are presented below, including the relative change to 2030 and an assessment of the reliability of the projections. The projections are compared with past trends to assess whether observed trends will continue.

Lithium consumption is projected to decrease in four countries (Table 4), including Denmark (− 2.7% relative change), Iceland (− 10.8%), Norway (− 10.3%) and Spain (− 2.3%). Six countries are expected to increase: Estonia (+ 19.2%), Finland (+ 17.2%), Germany (+ 4.1%), Italy (+ 20.0%), the Netherlands (3.4%) and Sweden (+ 2.5%). No model could be constructed for Croatia due to a lack of a sufficient number of data.

**Table 4:** Prediction of DID prescriptions with ARIMA models for lithium and antipsychotics until 2030. Last reported and predicted use, relative change and predicted trend are shown. Countries are sorted in descending order of last reported DID prescriptions of lithium

Country	Last reported DID prescriptions of lithium	Predicted DID prescriptions for lithium in 2030	Relative change lithium consumption	Predicted trend of lithium consumption	Last reported DID prescriptions of antipsychotics	Predicted DID prescriptions of antipsychotics in 2030	Relative change antipsychotics consumption	Predicted trend of antipsychotic consumption
Sweden	1.60	1.64	2.5%	Increase	9.85	10.41	5.7%	Increase
Norway	1.45	1.30	− 10.3%	Decrease	24.03	26.44	10.0%	Increase
Iceland	1.20	1.07	− 10.8%	Decrease	14.00	15.20	8.6%	Increase
Netherlands	1.17	1.21	3.4%	Increase	8.19	8.35	2.0%	Increase
Denmark	1.1	1.07	− 2.7%	Decrease	13.60	14.81	8.9%	Increase
Finland	0.99	1.16	17.2%	Increase	22.15	27.32	23.3%	Increase
Spain	0.86	0.84	− 2.3%	Decrease	14.50	16.45	13.4%	Increase
Germany	0.74	0.77	4.1%	Increase	12.37	13.61	10.0%	Increase
Italy	0.40	0.48	20.0%	Increase	10.60	12.31	16.1%	Increase
Croatia	0.26	Model cannot be built	-	-	16.05	20.34	26.7%	Increase
Estonia	0.26	0.31	19.2%	Increase	10.80	13.33	23.4%	Increase

Antipsychotic use is forecast to increase in all countries, including Croatia (+ 26.7%), Denmark (+ 8.9%), Estonia (+ 23.4%), Finland (+ 23.3%), Germany (+ 10.0%), Iceland (+ 8.6%), Italy (+ 16.1%), the Netherlands (+ 2.0%), Norway (+ 10.0%), Spain (+ 13.4%) and Sweden (+ 5.7%).

The reliability of the forecasts is generally rated as good or moderate (Table S8). The range of confidence intervals, represented by the upper and lower 95% confidence limits (UCL and LCL), is mostly narrow (less than 100% relative range). However, exceptions include the lithium forecasts in Estonia (158.1%) and Italy (152.1%), which show a moderate range, and Spain (216.7%), where the range is considerably wider. A narrow confidence interval indicates greater certainty in the forecasts, while a wider range reflects greater uncertainty.

In terms of model fit, the predictions for antipsychotic use are more reliable than those for lithium. This is probably due to a more continuous increasing trend (Bindel and Seifert 2025a, b, c) in antipsychotic use. The limited availability of data also affects the predictive power of the model (Bindel and Seifert 2025a, b, c). While stationary *R*-squared values are generally poor, overall *R*-squared values and forecast errors are predominantly good or moderate. High reliability and good fit metrics are observed for the lithium forecasts for Norway and for the antipsychotic forecasts for Croatia, Denmark, Estonia, Italy, Norway, Spain and Sweden. These forecasts are therefore considered to be robust. Moderate fit and reliability are found for the lithium and antipsychotic forecasts for Denmark, Finland, Germany, Iceland and the Netherlands, and for lithium consumption in Sweden. A moderate classification suggests that the general trend is likely to be correct, although individual values may deviate from actual future observations. Conversely, poor fit metrics and low reliability characterise the lithium forecasts for Estonia, Italy and Spain, with increased uncertainty reflected in their confidence intervals. However, poor reliability does not necessarily indicate incorrect predictions, but rather a high degree of uncertainty in the estimated values (Bindel and Seifert 2025a, b, c).

Overall, the projected changes in lithium consumption remain relatively small, with modest increases or decreases. For example, in Italy, a relative increase of 20.0% corresponds to an increase in DID prescriptions from 0.4 to 0.48. In contrast, the expected increase in antipsychotic use is more pronounced, as illustrated by Croatia, where prescriptions are expected to increase from 16.05 to 20.34. In all countries, the increase in the use of antipsychotics exceeds that of lithium, reinforcing the established trends of decreasing lithium prescriptions and confirming the observation of increasing substitution of lithium by antipsychotics.

### Outstanding countries and identification of rational and problematic prescribing behaviour

Countries show different prescribing patterns when considering DID prescriptions, lithium treatment coverage and the ratio of lithium to antipsychotic use. As these analyses were chosen to assess prescribing behaviour, countries with highly pronounced characteristics were examined to understand the underlying mechanisms. Based on these observations, general patterns were discussed to determine which characteristics favour rational lithium use and which suggest prescribing challenges. Countries were categorised as having good, moderate or poor prescribing behaviour, in comparison with each other (Table 5).

**Table 5:**
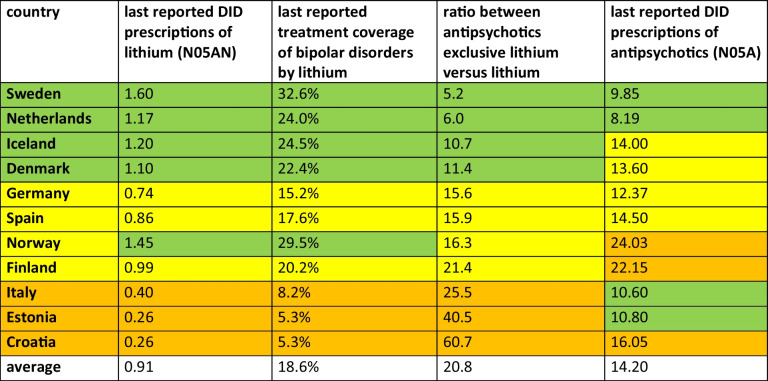
Assessment of lithium prescribing behaviour by comparing key characteristics of the 11 countries analysed. For each country, the DID prescriptions of lithium and its treatment coverage for bipolar disorder, the ratio of antipsychotics to lithium, and the DID prescriptions of antipsychotics are considered. A high number of DID prescriptions of lithium above 1 DID, a high treatment coverage above 22% and a low ratio below 12 are considered to be good prescribing practices. Moderate prescribing is considered to be 0.5–1.0 DID of lithium, 15–22% treatment coverage and a ratio of 12–22. Poor prescribing is defined as lithium use below 0.5 DID, treatment coverage below 10% and a ratio above 22. The DID of antipsychotic prescriptions has been used to assess lithium use in order to better understand the general level of use of pharmacological treatment. While a DID of less than 12 is considered relatively low, a DID of 12–16 is considered average and a DID of more than 16 is considered relatively high. Countries are coloured by their assessment of good, moderate and poor practice and ranked in ascending order of the ratio of antipsychotic use to lithium use

Notably, countries with one prominent feature often exhibit another. Estonia and Croatia have the lowest DID prescriptions and treatment coverage (0.26 DID and 5.3%) and the highest ratio of antipsychotics to lithium (60.7 for Croatia and 40.5 for Estonia). In contrast, Sweden and Norway have the highest DID prescriptions and lithium treatment coverage (1.6 DID and 32.6% for Sweden; 1.5 DID and 29.5% for Norway). Sweden and the Netherlands have the lowest ratios, with 5.2 and 6.0 respectively. For DID prescriptions of antipsychotics, Norway (24.0), Finland (22.2) and Croatia (16.1) have the highest consumption, while the Netherlands (8.2) and Sweden (9.9) have the lowest.

A relatively high lithium consumption and a low ratio (antipsychotics vs. lithium consumption) are preferable, as they indicate a more rational use of lithium. Conversely, low DID prescriptions and a high ratio suggest that lithium is being frequently replaced by other antipsychotics. Analysis of antipsychotic DID prescriptions helps to contextualise lithium use and highlights potential discrepancies in prescribing behaviour.

Countries with comparatively good lithium prescription patterns include the Netherlands, Sweden, Denmark and Iceland (Tables 5 and 6). Sweden and the Netherlands stand out for their high lithium use, low ratio and low antipsychotic use. Denmark and Iceland also have high lithium use but moderate antipsychotic use. In all these countries, however, lithium use has plateaued while antipsychotic use has increased. The literature supports this grouping. In the Netherlands, lithium is widely used for bipolar disorder, suggesting strong adherence to guidelines (Pérez de Mendiola et al. 2021), with national specialist programmes addressing some adherence gaps (Renes et al. 2014). Sweden has a national quality registry for bipolar disorder (BipoläR), which is improving the quality of treatment (Sköld et al. 2021). Similarly, Denmark strongly promotes lithium over antipsychotics for bipolar disorder (Danish Health Authority 2016). No specific information was found for Iceland.

**Table 6:**
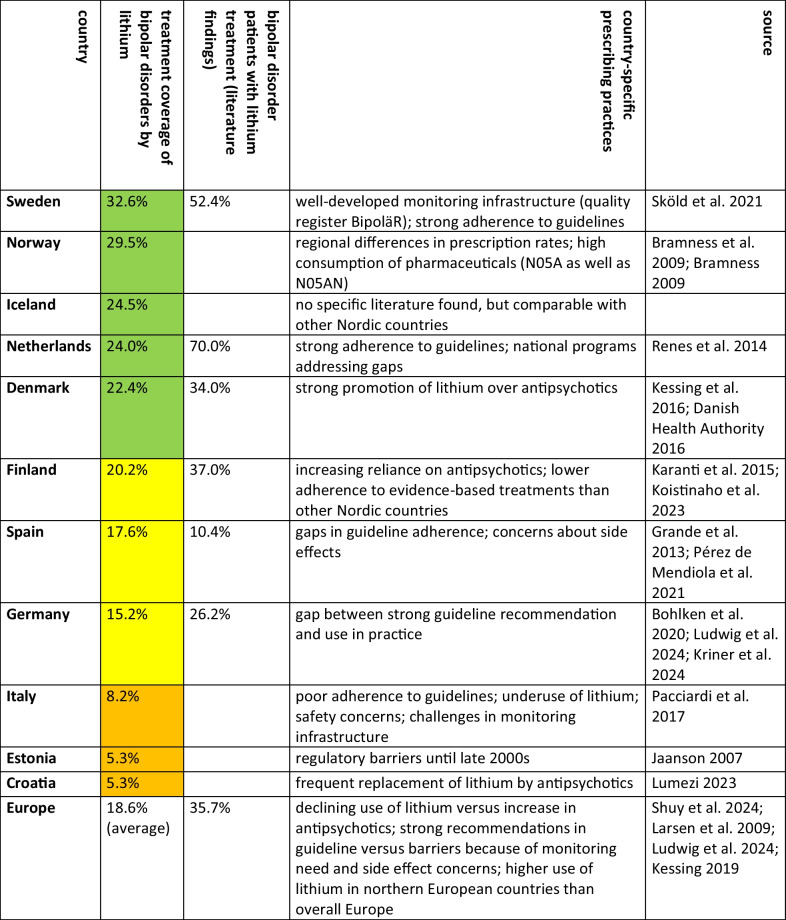
Characterisation of prescribing practices in the countries analysed. In addition, the calculated treatment coverage of the prevalence of bipolar disorder is shown, as well as the proportion of patients treated with lithium found in the literature. The treatment coverage of the prevalence of bipolar disorder with lithium is highlighted in colour, with green indicating an above-average proportion, yellow an average proportion and orange a below-average proportion. This classification is consistent with the characterisation of prescribing behaviour. Countries are sorted in descending order of treatment coverage of bipolar disorder prevalence

Countries with moderate lithium use include Germany, Spain, Finland and Norway (Tables 5 and 6). These countries have a moderate ratio of antipsychotics to lithium. Norway stands out with high lithium use, which is explained by its generally high rates of pharmacological treatment rather than strict adherence to lithium guidelines. Lithium use has plateaued in these countries, while antipsychotic use has increased. Norway’s lithium use is in line with other Nordic countries (Bramness et al. 2009), although regional differences suggest uneven guideline adoption (Bramness 2009). Finland has a lower use of evidence-based treatments and an increasing reliance on antipsychotics (Koistinaho et al. 2023). In Spain, 70% of psychiatrists adhere to guidelines (Pérez de Mendiola et al. 2021), but side effect concerns lead to lower prescribing of lithium, especially in younger patients. In Germany, lithium is strongly recommended in guidelines (Ludwig et al. 2024), but its use remains low due to a gap between recommendations and practice (Kriner et al. 2024).

Countries with poor lithium prescription behaviour include Italy, Croatia and Estonia (Tables 5 and 6). These countries have low lithium use and a high ratio of antipsychotics to lithium. While Italy and Estonia have a comparably low consumption of antipsychotics, Croatia has a high rate of use, exacerbating its unbalanced prescribing pattern. Croatia and Estonia stand out for their low lithium use and high ratio. Antipsychotic use has risen sharply in Croatia and Estonia, while there has been a slight increase in Italy. However, lithium prescriptions have also increased slightly over time in these countries. The literature confirms poor adherence to guidelines in Italy, with significant underuse of lithium (Pacciardi et al. 2017). Croatia also shows poor adherence, with lithium being replaced by atypical antipsychotics due to concerns about side effects and monitoring requirements (Lumezi 2023). Prescribing patterns in Estonia are influenced by regulatory barriers, as lithium was not registered as a therapeutic agent until the late 2000 s (Jaanson 2007), hinder its integration into clinical practice.

In summary, rational treatment of bipolar disorder with lithium is characterised by lithium consumption and the ratio of antipsychotics to lithium, as well as antipsychotic consumption to identify potential discrepancies. The literature supports the country categorisations: the Netherlands, Sweden, Denmark and Iceland show good prescribing behaviour; Germany, Spain, Finland and Norway show moderate behaviour; and Italy, Croatia and Estonia show poor adherence to guidelines. Differences in prescribing behaviour are due to efforts to implement guidelines, distorted perception of safety concerns and practicability, clinician training and prescribing preferences. Regarding DDD costs, there is no pattern recognisable, meaning that countries with good prescribing behaviour have comparably high costs and others with poor practice can have low costs (Table 7). In overall, differences in prescribing practice cannot be fully explained by rational factors alone and require further evaluation.

**Table 7:**
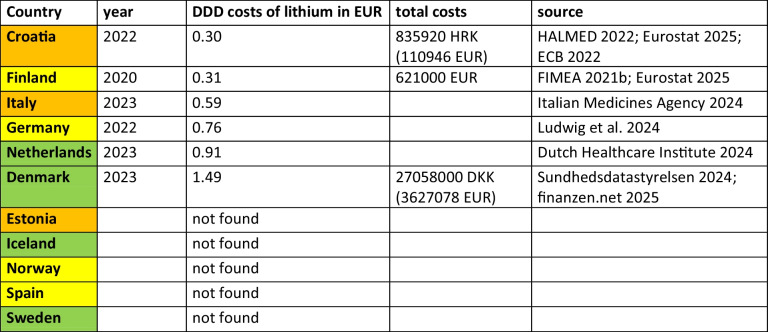
Analysis of DDD costs in € of lithium (ATC N05AN) for the countries analysed. Year and total costs are given. Countries are coloured according to their assessed prescribing behaviour, with green representing good practice, yellow moderate practice and orange poor practice. Countries are sorted in ascending order of DDD costs

### Geographical distribution and shifts in prescribing behaviour

Significant differences in lithium prescribing patterns exist between individual countries, yet certain regional similarities can also be identified. To facilitate a broader generalisation and a deeper understanding of these prescribing patterns, countries have been categorised into Northern and Southern European regions. Northern Europe includes the Netherlands, Sweden, Denmark, Iceland, Germany, Finland, Norway and Estonia, while Southern Europe includes Spain, Italy and Croatia. To compare differences between these regions, the mean DID prescriptions of lithium, its treatment coverage for bipolar disorder, the ratio of antipsychotic to lithium use and the DID prescriptions of antipsychotics were analysed (Table 8).

**Table 8:**
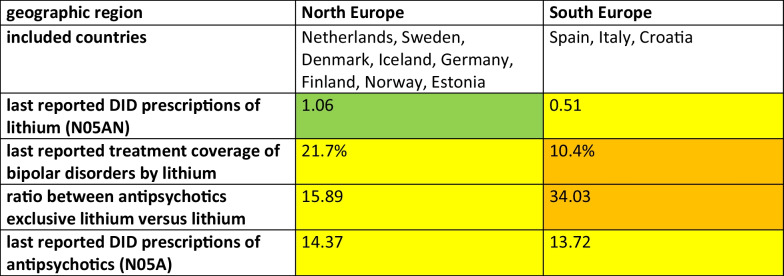
Assessmentof prescribing behaviour by geographical region of Northern and Southern Europe for the countries analysed. For each region, the mean DID prescriptions of lithium and its treatment coverage for bipolar disorder, the ratio of antipsychotics to lithium and the DID prescriptions of antipsychotics are considered. A high number of lithium DID prescriptions above 1 DID, a high treatment coverage above 22% and a low ratio below 12 are considered good prescribing practices. Moderate prescribing is defined as 0.5–1.0 DID of lithium, 15–22% treatment coverage and a ratio of 12–22. Poor prescribing is defined as lithium use below 0.5 DID, treatment coverage below 10% and a ratio above 22. The DID of antipsychotic prescriptions was used to assess lithium use in order to better understand the general level of use of pharmacological treatment. While a DID of less than 12 is considered relatively low, a DID of 12–16 is considered average and a DID of more than 16 is considered relatively high

In terms of recent DID prescriptions of lithium, Northern Europe has an average of 1.06 DID, whereas Southern Europe has only 0.51 DID. This discrepancy is also reflected in the treatment coverage of bipolar disorder with lithium, which is 21.7% in Northern Europe compared with 10.4% in Southern Europe. The ratio of antipsychotics to lithium is 15.9 in Northern Europe and significantly higher in Southern Europe, at 34.0. The difference in antipsychotic use is minimal, with 14.4 DID in Northern Europe and 13.7 DID in Southern Europe.

These substantial regional differences in lithium prescribing cannot be explained by differences in the pharmacological treatment of mental disorders or the prevalence of bipolar disorder, as these factors remain relatively similar in both regions. The influence of temperature and sunlight is discussed, with some studies finding an effect and others not (Bauer et al. 2009, 2012). However, the impact remains minor. Instead, the observed discrepancies strongly suggest differences in prescribing behaviour (Table 6). The literature confirms this north–south trend, indicating that lithium is prescribed more frequently in Northern Europe than in Southern Europe (Larsen et al. 2009) (Fig. 3). Although this categorisation simplifies the regional divide, there is also evidence of a west–east trend, with lower lithium use in Eastern Europe (Vieta et al. 2013). This trend is evident in the data analysed, as Estonia and Croatia, both more eastern countries, have exceptionally low lithium prescriptions.

Several factors may contribute to these differences in prescribing behaviour. Healthcare infrastructure plays a crucial role. In northern European countries such as Sweden, a well-developed infrastructure ensures fewer side effects and better adherence to treatment (Golic 2018; Sköld et al. 2021). Conversely, in countries such as Italy, where the healthcare infrastructure is less developed, lithium treatment may result in more side effects and higher safety risks (Pacciardi et al. 2017). However, it is important to be aware that antipsychotics require monitoring to a similar extent as lithium. While these monitoring requirements are often met or exceeded (Strejilevich et al. 2011), this is not the case for antipsychotics (Azfr Ali et al. 2021).

Clinical training and cultural attitudes towards lithium use also influence prescribing practices. In Northern Europe, where lithium use has a long history (Shorter 2009), it is more widely accepted. In contrast, countries such as Estonia, which have historically relied on alternative treatments (Jaanson 2007), have less experience with lithium, leading to greater scepticism. Concerns about side effects also limit the use of lithium. In addition, lithium is a generic and older drug, leading to minimal financial incentives for marketing (Airainer and Seifert 2024). In contrast, antipsychotics have been heavily promoted (Apollonio 2022), increasing their perceived availability and prescription rates (Fickweiler et al. 2017). Physicians in certain regions, particularly in south-eastern Europe, may be more susceptible to marketing influences (Fadlallah et al. 2018), leading to a greater shift towards prescribing antipsychotics over lithium compared to countries with stricter regulations. Another key factor is adherence to treatment guidelines. Northern European countries generally show higher adherence to evidence-based prescribing guidelines (Pérez de Mendiola et al. 2021), whereas southern European countries show lower adherence (Pacciardi et al. 2017; Lumezi 2023). Although there is a slightly greater emphasis on the use and role of lithium in the northern European regions, the differences in guidelines are not large enough to explain the differences in prescribing behaviour (Table 9).

**Table 9:** Comparison of guidelines between countries in different regions, exemplarily for Sweden, Germany and Spain. Certain aspects of lithium use are mentioned and compared for their similarities and differences

**Criteria**	Sweden (Socialstyrelsen 2021)	Germany (DGBS and DGPPN 2019)	Spain (Ministerio de Sanidad 2020, 2023)	Similarities	Differences
Considered prescribing practice of lithium	Good	Moderate	Moderate	Sweden and Germany North Europe; Germany and Spain moderate prescribing behaviour	Differences in considerations of rational use; differences in geographical region
Indication of lithium	Bipolar I & II, long-term prevention	Bipolar I & II, long-term prevention	Bipolar I, less use in Bipolar II	First-line for bipolar disorder	Less emphasis on lithium for Bipolar II in Spain
Adherence to lithium as first-line treatment	High adherence, preferred first-line	Moderate adherence, frequent alternatives	Low adherence, often used in combination therapy	Lithium as first-line treatment in all	Higher lithium adherence in Sweden than Spain
Monitoring and safety regulations	Regular TDM, BipoläR registry	Routine TDM, focus on renal function	Regular TDM, monitoring of kidney and thyroid function recommended​	Lithium monitoring required	Sweden has the most structured monitoring
Use in acute vs. maintenance therapy	Mainly recommended for long-term mood stabilisation, less commonly used as a primary agent in acute manic episodes, some acute use	Used in both acute mania and maintenance, flexible approach	Mainly maintenance, less frequent in acute mania​	Lithium used for long-term stabilisation	Germany uses lithium more often for acute mania than Sweden/Spain
Alternative treatment preferences	Low reliance on valproate/antipsychotics	Frequent use of valproate/lamotrigine	Frequent use of valproate and lamotrigine instead of lithium	Valproate as an alternative	Spain is more flexible in using valproate/lamotrigine
Suicide prevention focus	Strong, emphasised in guidelines	Strong, acknowledged	Strong, emphasised, particularly in high-risk patients	Lithium recognised for suicide prevention	No major differences
Structural support and implementation	BipoläR registry, high adherence	No registry, adherence moderate	No registry, but structured monitoring required	All recommend lithium use	Sweden has the strongest support system
Special populations	Adjusted dosage for risk groups; not recommended in early pregnancy			Frequent monitoring required in all risk groups	Spain has more restrictions in pregnancy and severe renal/cardiac conditions

In summary, there are significant regional differences in lithium use, suggesting differences in prescribing behaviour. Northern European countries generally have a more evidence-based and rational prescribing approach compared with southern European countries. However, there are also variations within these broader regions, such as Estonia in Northern Europe, which has lower lithium use. These regional differences extend beyond antipsychotics, as similar trends are observed in the prescribing behaviour of other drug classes, including antibacterials (Bindel and Seifert 2025a, b) and thyroid hormones (Bindel and Seifert 2025c). Broader prescribing behaviours, such as rational drug use in general, also reflect systemic influences rather than individual prescriber preferences (Rotar et al. 2020; Tian et al. 2023). This underscores the presence of structural settings affecting prescribing patterns across regions.

## Limitations

This study is based on data from national health authorities in 11 European countries. An important limitation is that not all European countries were included, resulting in gaps and uncertainties in regional patterns. In addition, the observation periods for the available prescription data vary. While some countries provided data only for recent years, others published data covering several decades. Because of these differences in time periods, the comparison of past trends was limited to identifying trends rather than assessing absolute values of relative changes.

A key assumption in this study is that a patient’s dosage corresponds to a defined daily dose (DDD) prescription. However, in clinical practice, individual treatment dosages vary (Malhi and Tanious 2011). Nevertheless, DID are considered a valuable tool in the assessment of trends and international comparisons (WHO 2025). In addition, prevalence rates were assumed to be relatively similar across the countries analysed (GBD 2019 Mental Disorders Collaborators 2022). Differences in prevalence (Oliva et al. 2025) could lead to differences in treatment coverage. Therefore, the interpretation of the treatment coverages comes with uncertainties and is to consider in comparison with other countries rather than an exact value.

To classify countries into categories of good, moderate and poor prescribing behaviour, ranges were established based on DID prescriptions of lithium and antipsychotics, the ratio of antipsychotics to lithium and the treatment coverage of bipolar disorder with lithium. If these thresholds were changed, alternative categorisations and results might emerge.

Data availability was a challenge for the development of the forecasting models, with some countries reporting limited data points (Table 1). Certain countries were considered to have a low number of data but were included to ensure comprehensive regional coverage. A relatively short observation period required a simpler modelling approach. Although ARIMA models are widely used for forecasting time series, they assume linear relationships and may not fully capture complex patterns. Unexpected future developments cannot be fully anticipated, which means that long-term projections up to 2030 should be interpreted as indicative trends rather than exact values. Alternative statistical approaches, modifications to the model or adjustments to the fit metrics could lead to different assessments (Bindel and Seifert 2025a, b, c).

Lithium and, especially, antipsychotics are not limited to a single indication (Ludwig et al 2024). Their use extends to multiple conditions, including off-label use and polypharmacy (Ludwig et al. 2024). This wide range of indications makes it difficult to assess treatment coverage accurately and limits comparisons between lithium and antipsychotics.

## Conclusions and further perspectives

The analysis of lithium and antipsychotics was conducted for 11 European countries using publicly available prescription data (Table 1). Based on past and predicted DID prescriptions of lithium and antipsychotics, the coverage of bipolar disorder with lithium and the ratio of antipsychotics to lithium were assessed to evaluate prescribing patterns (Tables 2, 3, 4 and 5 and Figs. 2, 3 and 4). As lithium is considered the superior treatment for bipolar disorder (Ludwig et al. 2024; Shuy et al. 2024), high DID prescriptions and treatment coverages are considered favourable, while a low ratio between antipsychotics and lithium is preferred.

There is strong evidence of underuse of lithium in all European countries analysed, as indicated by treatment coverage of less than one third of the estimated prevalence of bipolar disorder (Fig. 3). Furthermore, a north–south shift is observed (Tables 5 and 8), with an additional west–east shift suggested. Nordic countries, including the Netherlands, Sweden, Denmark and Iceland, show relatively rational prescribing behaviour (Table 6). Other northern European countries, such as Germany, Finland, Norway and Estonia, show moderate adherence to guidelines. In contrast, Southern European countries, including Spain, Italy and Croatia, show poor lithium use. Despite regional differences, all European countries share the same trend: lithium use has plateaued or declined in recent years, while antipsychotic use has increased (Bohlken et al. 2020), resulting in an increasing ratio of antipsychotics to lithium. This trend is expected to continue in the future and is worrying because it suggests a lack of progress towards more rational pharmacological treatment, a trajectory also observed for antibacterial drugs (Bindel and Seifert 2025a, b).

It is of great concern that an effective, accessible and well-established treatment such as lithium for bipolar disorder remains underused (Shuy et al. 2024). Moreover, lithium is increasingly being replaced by poorly recommended alternatives such as antipsychotics (Samalin et al. 2016; Pérez de Mendiola et al. 2021), exacerbating its underuse and the overprescription of antipsychotics.

A frequently mentioned concern about lithium treatment is its potential toxicity and side effects (Ludwig et al. 2024). However, this concern seems paradoxical. Lithium’s long history of use has resulted in a well-documented and well-understood side effect profile (Gitlin 2016). This extensive knowledge may contribute to increased awareness among clinicians, potentially leading to restricted use of lithium. In contrast, antipsychotics have a broader and less predictable range of severe side effects, with long-term effects that are not yet fully understood (Allott et al. 2024). Lithium toxicity can be managed in an effective way by monitoring, which antipsychotics also require (Oliva et al. 2025). Countries such as Sweden are demonstrating how structured monitoring can reduce side effects and improve adherence in lithium treatment (Sköld et al. 2021). These misconceptions must be addressed in the education and training of professionals.

It is important to recognise that treatment choices have a significant impact on patient outcomes and health system costs. Mental disorders are among the top ten leading causes of disease burden worldwide, requiring effective prevention and treatment (GBD 2019 Mental Disorders Collaborators 2022). The lack of reduction in the burden of disease since 1990 (GBD 2019 Mental Disorders Collaborators 2022) is consistent with the lack of observed improvements in the treatment of bipolar disorder. The superiority of lithium in preventing mood relapse and reducing suicide risk in bipolar patients is well documented (Kessing 2019). Regions with higher lithium use have significantly lower recurrence rates of bipolar episodes (Sköld et al 2021), which in turn reduces hospitalisations and associated healthcare costs (Ekman et al 2013). Lithium is also a cost-effective treatment option (Airainer and Seifert 2024). Failure to adhere to treatment guidelines can lead to manic episodes, rapid cycling and worsening disease course (Vieta et al. 2013), with serious consequences for patients and a substantial financial burden on healthcare systems (Ekman et al. 2013). Conversely, side effects of antipsychotics like metabolic syndromes generate a high cost-of-illness (Jerrell et al. 2012). Withholding lithium from eligible patients is therefore both medically and economically irresponsible.

Further research is needed to validate these findings, to examine additional influencing factors and to extend the analysis to other countries, regions and continents for a more comprehensive global understanding. In addition, other drug classes should be examined to identify rational and irrational prescribing behaviour at the national level and regional shifts in drug use patterns.

### Take-home messages

- Lithium is the superior first-line treatment for bipolar disorders.
- Alarmingly, lithium is significantly underused across all analysed European countries, with a north–south shift in prescribing behaviour.
- There are no clinical reasons for the underuse of lithium.
- Widespread misconceptions persist regarding the adverse effects and monitoring efforts of lithium, with antipsychotics being recognised as safer and easier to handle. This is not the case, as antipsychotics carry a range of severe and less predictable adverse effects and require similar monitoring. In contrast, the adverse effects of lithium are well-documented, making its management more predictable and safer.
- Lithium is the drug with adequate cost-effectiveness in the long-term treatment of bipolar disorders.
- Improving bipolar disorder treatment requires increased lithium use. This requires educational initiatives for healthcare professionals and efforts to promote the use of lithium while reducing the influence of pharmaceutical promotion for antipsychotics.

## Supplementary Information

Below is the link to the electronic supplementary material.

ESM 1 (DOCX 53.0 KB)

## Data Availability

All source data for this study are available upon reasonable request from the authors.

## Abbreviations

ACF: Autocorrelation function
ARIMA: Auto Regressive Integrated Moving Average model (mathematical model for time series analysis)
ATC: Anatomical Therapeutic Chemical Classification System (drug classification system, developed by the WHO; https://atcddd.fhi.no/atc_ddd_index/)
DDD: Defined Daily Dose prescriptions
DID: Defined Daily Dose prescriptions per 1000 inhabitants per day
GBD: Global Burden of Disease study (https://www.thelancet.com/gbd)
LCL: Lower confidence limit
N05A: ATC code for antipsychotic drugs
N05AN: ATC code for lithium
PACF: Partial autocorrelation function
SPSS: Statistical Product and Service Solutions (software for statistical analyses, IBM)
UCL: Upper confidence limit

## References

[CR1] Agency for Medicinal Products and Medicals Devices (HALMED) (2014) Potrošnja lijekova u Hrvatskoj 2007 - 2012. godine Last updated: 2014. Last accessed: 18 Feb 2025. https://www.halmed.hr/fdsak3jnFsk1Kfa/publikacije/Potrosnja_lijekova_u_Hrvatskoj_2007-2012.pdf

[CR2] Agency for Medicinal Products and Medicals Devices (HALMED) (2023) Potrošnja lijekova u Hrvatskoj 2018 – 2022. Last updated: 2023. Last accessed: 18 Feb 2025. https://www.halmed.hr/fdsak3jnFsk1Kfa/publikacije/Potrosnja-lijekova-u-Hrvatskoj-2018-2022.pdf

[CR3] Agency for Medicinal Products and Medicals Devices (HALMED) (2022) UKUPNA POTROŠNJA LIJEKOVA U 2022. GODINI. Last updated: 2022. Last accessed: 06 March 2025. https://www.halmed.hr/fdsak3jnFsk1Kfa/ostale_stranice/Tablica_16-Ukupna_potrosnja_lijekova_u_2022_godini.pdf

[CR4] Agency for Medicinal Products and Medicals Devices (HALMED) (2018) Potrošnja lijekova u Hrvatskoj 2013 – 2017. Last updated: 2018. Last accessed: 18 Feb 2025. https://www.halmed.hr/fdsak3jnFsk1Kfa/publikacije/Potrosnja-lijekova-u-Hrvatskoj-2013-2017.pdf

[CR5] Airainer M, Seifert R (2024) Lithium, the gold standard drug for bipolar disorder: analysis of current clinical studies. Naunyn-Schmiedeberg’s Arch Pharmacol 397(12):9723–9743. 10.1007/s00210-024-03210-8 38916833 10.1007/s00210-024-03210-8 PMC11582333

[CR6] Allott K, Chopra S, Rogers J et al (2024) Advancing understanding of the mechanisms of antipsychotic-associated cognitive impairment to minimise harm: a call to action. Mol Psychiatry 29:2571–2574. 10.1038/s41380-024-02503-x 38454078 10.1038/s41380-024-02503-x PMC11412898

[CR7] Apollonio DE (2022) Marketing antipsychotics to correctional facilities: a review of pharmaceutical industry documents. J Correct Health Care 28(5):325–328. 10.1089/jchc.21.04.0026 36190495 10.1089/jchc.21.04.0026 PMC9835282

[CR8] Azfr Ali RS, Jalal Z, Paudyal V (2021) Guidelines versus practice in screening and monitoring of cardiometabolic risks in patients taking antipsychotic medications: where do we stand? Gen Psychiatry 34(4). 10.1136/gpsych-2021-100561 10.1136/gpsych-2021-100561 PMC8311327 34396043

[CR9] Bauer M, Glenn T, Alda M, Andreassen OA, Ardau R, Bellivier F, Berk M, Bjella TD, Bossini L, Del Zompo M, Dodd S, Fagiolini A, Frye MA, Gonzalez-Pinto A, Henry C, Kapczinski F, Kliwicki S, König B, Kunz M, Lafer B, … Whybrow PC (2012) Impact of sunlight on the age of onset of bipolar disorder. Bipolar Disord 14(6):654–663. 10.1111/j.1399-5618.2012.01025.x 10.1111/j.1399-5618.2012.01025.x PMC3525652 22612720

[CR10] Bauer M, Glenn T, Grof P, Rasgon NL, Marsh W, Sagduyu K, Alda M, Murray G, Quiroz D, Malliaris Y, Sasse J, Pilhatsch M, Whybrow PC (2009) Relationship among latitude, climate, season and self-reported mood in bipolar disorder. J Affect Disord 116(1–2):152–157. 10.1016/j.jad.2008.11.013 19091424 10.1016/j.jad.2008.11.013

[CR11] Bindel LJ, Seifert R (2025a) Most European countries will miss EU targets on antibacterial use by 2030: historical analysis of European and OECD countries, comparison of community and hospital sectors and forecast to 2040. Naunyn-Schmiedeberg’s Arch Pharmacol. 10.1007/s00210-025-03887-5 10.1007/s00210-025-03887-5 PMC12350445 39960558

[CR12] Bindel LJ, Seifert R (2025b) AWaRe classification analysis for European countries with ARIMA forecasts to assess prescribing patterns and ‘One Health’ targets. Naunyn-Schmiedeberg's Arch Pharmacol. 10.1007/s00210-025-04121-y 10.1007/s00210-025-04121-y PMC12511250 40220024

[CR13] Bindel LJ, Seifert R (2025c) Long-term forecasting and evaluation of medicine consumption for the ATC class H with a focus on thyroid hormones in OECD countries using ARIMA models. Naunyn-Schmiedeberg's Arch Pharmacol. 10.1007/s00210-025-03930-5 10.1007/s00210-025-03930-5 PMC12350577 40029386

[CR14] Bohlken J, Bauer M, Kostev K (2020) Drug treatment for patients with bipolar disorders in psychiatric practices in Germany in 2009 and 2018. Psychiatry Res 289:112965. 10.1016/j.psychres.2020.112965 32388174 10.1016/j.psychres.2020.112965

[CR15] Bramness JG (2009) Bruk av litium i Oslo og i Sogn og Fjordane [Use of lithium in the Norwegian counties Oslo and Sogn og Fjordane]. Tidsskrift for Den Norske Laegeforening : Tidsskrift for Praktisk Medicin, Ny Raekke 129(9):855–857. 10.4045/tidsskr.08.0276 19415083 10.4045/tidsskr.08.0276

[CR16] Bramness JG, Weitoft GR, Hallas J (2009) Use of lithium in the adult populations of Denmark, Norway and Sweden. J Affect Disord 118(1–3):224–228. 10.1016/j.jad.2009.01.024 19249102 10.1016/j.jad.2009.01.024

[CR17] Caetano J, Basso LA, de Lima Argimon II, Arteche A (2013) Systematic review of the prevalence of bipolar disorder and bipolar spectrum disorders in population-based studies. Trends Psychiatry Psychother 35(2). 10.1590/S2237-60892013000200002 10.1590/s2237-60892013000200002 25923299

[CR18] Castaldelli-Maia JM, Bhugra D (2022) Analysis of global prevalence of mental and substance use disorders within countries: focus on sociodemographic characteristics and income levels. Int Rev Psychiatry 34(1):6–15. 10.1080/09540261.2022.2040450 35584016 10.1080/09540261.2022.2040450

[CR19] Cipriani A, Hawton K, Stockton S, Geddes JR (2013) Lithium in the prevention of suicide in mood disorders: updated systematic review and meta-analysis. BMJ (Clinical Research Ed) 346:f3646. 10.1136/bmj.f3646 23814104 10.1136/bmj.f3646

[CR20] Cookson J (2001) Use of antipsychotic drugs and lithium in mania. Br J Psychiatry 178(S41):s148–s156. 10.1192/bjp.178.41.s148 11388955

[CR21] Danish Health Authority (2016) National clinical guideline for the pharmacological treatment of bipolar disorder – add-on maintenance treatment following depression. Quick guide. Last updated: July 2016. Last accessed: 26 Feb 2025. https://www.sst.dk/-/media/Udgivelser/2014/NKR-Medicinsk-behandling-af-bipolar-lidelse/Nr--8-Quickguide-Bipolar-lidelse.ashx?sc_lang=da&hash=BBA17F7D8DD8245A14C440DD3CCF33B8#:~:text=%E2%86%91%20Consider%20initiating%20maintenance%20treatment,Health%20Authority%20recommends%20for%20lamotrigine

[CR22] Deutsche Gesellschaft für Bipolare Störungen, Deutsche Gesellschaft für Psychiatrie und Psychotherapie, Psychosomatik und Nervenheilkunde (DGBS, DGPPN) (2019) S3-Leitlinie zur Diagnostik und Therapie Bipolarer Störungen. Langversion 2.1. AWMF-Register Nr. 038–019. Last updated: May 2020. Last accessed: 04 March 2025. https://www.dgppn.de/_Resources/Persistent/ef9214009e20d260d4f5a6e6932f3fb7f914efbb/S3_Leitlinie%20Bipolar_V2.1_Update_20200504.pdf

[CR23] Dickey DA, Fuller WA (1979) Distribution of the estimators for autoregressive time series with a unit root. J Am Stat Assoc 74(366):427–431. 10.2307/2286348

[CR24] Directorate of Health (2025) Lyfjanotkun á Íslandi. Eftir ATC flokkunarkerfi lyfja. Last accessed: 19 Feb 2025. Last updated: January 2025. https://app.powerbi.com/view?r=eyJrIjoiZmRiMGJkNmMtZWQ4NC00NmUzLTlkY2UtZTQ0NDk5ZjZmMDE2IiwidCI6Ijc2NGEzMDZkLTBhNjgtNDVhZC05ZjA3LTZmMTgwNDQ0N2NkNCIsImMiOjh9

[CR25] Dutch Healthcare Institute (2024) GIPdatabank.nl. Last updated: May 2024. Last accessed: 18 Feb 2025. https://www.gipdatabank.nl/databank?infotype=g&label=00-totaal&tabel=B_01-basis&geg=ddd&item=N

[CR26] Ekman M, Ola G, Sead O, Johanna J, Mikael L (2013) The societal cost of bipolar disorder in Sweden. Soc Psychiatry Psychiatr Epidemiol. https://doi.org/48. 10.1007/s00127-013-0724-9 10.1007/s00127-013-0724-9 23754681

[CR27] Escamilla MA, Zavala JM (2008) Genetics of bipolar disorder. Dialogues Clin Neurosci 10(2):141–152. 10.31887/DCNS.2008.10.2/maescamilla 10.31887/DCNS.2008.10.2/maescamilla PMC3181866 18689285

[CR28] Estonian State Agency of Medicines (2013) Baltic statistics on medicines 2010–2012. Tartu, Estonia. ISBN 978–9949–33–397–4. Last accessed: 18 Feb 2025. Last updated: 2013. https://www.zva.gov.lv/sites/default/files/2018-05/BS_2013.pdf

[CR29] European Central Bank (ECB) (2022) Pressemitteilung. Kroatien tritt Euroraum am 1. Januar 2023 bei. Last updated: July 2022. Last accessed: 06 March 2024. https://www.ecb.europa.eu/press/pr/date/2022/html/ecb.pr220712~b97dd38de3.de.html

[CR30] Eurostat (2025) Database. Population (national level). European Union. Last updated: February 2025. Last accessed: 06 March 2025. https://ec.europa.eu/eurostat/databrowser/view/demo_pjan/default/table?lang=en&category=demo.demo_pop

[CR31] Fadlallah R, Alkhaled L, Brax H, Nasser M, Rajabbik MH, Nass H, A Kahale L, A Akl E (2018) Extent of physician–pharmaceutical industry interactions in low- and middle-income countries: a systematic review. Eur J Public Health 28(2):224–230. 10.1093/eurpub/ckx204 10.1093/eurpub/ckx204 29165586

[CR32] Fickweiler F, Fickweiler W, Urbach E (2017) Interactions between physicians and the pharmaceutical industry generally and sales representatives specifically and their association with physicians’ attitudes and prescribing habits: a systematic review. BMJ Open 7(9):e016408. 10.1136/bmjopen-2017-016408 28963287 10.1136/bmjopen-2017-016408 PMC5623540

[CR33] finanzen.net (2025) Währungsrechner. Dänische Krone - Euro. Last updated: March 06 2025. Last accessed: 06 March 2025. https://www.finanzen.net/waehrungsrechner/daenische-krone_euro?amount=1&date=2025-03-06&interbankrate=0

[CR34] Finnish Medicines Agency (FIMEA) (2021a) Drug consumption statistics. Drug consumption in years 2018–2021. Last accessed: 19 Feb 2025. http://raportit.nam.fi/raportit/kulutus/laakekulutus_e.html

[CR35] Finnish Medicines Agency (FIMEA) (2021b) Drug consumption statistics. Drug sales in years 2018–2021. Last accessed: 06 March 2025. http://raportit.nam.fi/raportit/kulutus/laakemyynti_e.pdf

[CR36] Folkehelseinstituttet (2021) Norwegian Prescription Database. The Norwegian Institute of Public Health. Last updated: April 2021. Last accessed: 20 Feb 2025. https://www.norpd.no/

[CR37] Folkehelseinstituttet (2024) Drug consumption in Norway 2019–2023. Data from Norwegian Drug Wholesales Statistics. The Norwegian Institute of Public Health. Oslo, Norway. ISBN: 978–82–8406–459–8. Last updated: June 2024. Last accessed: 20 Feb 2025. https://www.fhi.no/contentassets/b0802ad9303347b682cf6a8fa701ec91/legemiddelforbruket-i-norge-2019-2023-rapport-2024.pdf

[CR38] GBD 2019 Mental Disorders Collaborators (2022) Global, regional, and national burden of 12 mental disorders in 204 countries and territories, 1990–2019: a systematic analysis for the Global Burden of Disease Study 2019. Lancet Psychiatry 9(2):137–150, ISSN 2215–0366. 10.1016/S2215-0366(21)00395-3 10.1016/S2215-0366(21)00395-3 PMC8776563 35026139

[CR39] Gitlin M (2016) Lithium side effects and toxicity: prevalence and management strategies. Int J Bipolar Disord 4(1):27. 10.1186/s40345-016-0068-y 27900734 10.1186/s40345-016-0068-y PMC5164879

[CR40] Gitlin M, Bauer M (2024) Lithium: current state of the art and future directions. Int J Bipolar Disord 12:40. 10.1186/s40345-024-00362-7 39609318 10.1186/s40345-024-00362-7 PMC11604892

[CR41] Golic M, Aiff H, Attman PO, Ramsauer B, Schön S, Svedlund J (2018) Compliance with the safety guidelines for long-term lithium treatment in Sweden. J Psychopharmacol (Oxford, England) 32(10):1104–1109. 10.1177/0269881118780014 10.1177/0269881118780014 29896998

[CR42] Grande I, de Arce R, Jiménez-Arriero MÁ, Lorenzo FG, Valverde JI, Balanzá-Martínez V, Zaragoza S, Cobaleda S, Vieta E, and SIN-DEPRES Group (2013) Patterns of pharmacological maintenance treatment in a community mental health services bipolar disorder cohort study (SIN-DEPRES). Int J Neuropsychopharmacol 16(3):513–523. 10.1017/S1461145712000405 22717099 10.1017/S1461145712000405

[CR43] Greil W, de Bardeci M, Nievergelt N et al (2025) Twenty-four years of prescription patterns in bipolar disorder inpatients with vs without lithium: a pharmacoepidemiological analysis of 8,707 cases in German-speaking countries. Int J Bipolar Disord 13:3. 10.1186/s40345-025-00370-1 39945975 10.1186/s40345-025-00370-1 PMC11825962

[CR44] Gurung R, Prata DP (2015) What is the impact of genome-wide supported risk variants for schizophrenia and bipolar disorder on brain structure and function? A Systematic Review Psychol Med 45(12):2461–2480. 10.1017/S0033291715000537 25858580 10.1017/S0033291715000537

[CR45] Hálfdánarson Ó, Zoëga H, Aagaard L, Bernardo M, Brandt L, Coma Fusté A, Furu K, Garuoliené K, Hoffmann F, Huybrechts KF, Kalverdijk LJ, Kawakami K, Kieler H, Kinoshita T, Litchfield M, López SC, Machado-Alba JE, Machado-Duque ME, Mahesri M, Nishtala PS, Pearson S-A, Reutfors J, Saastamoinen LK, Sato I, Schuiling-Veninga CCM, Shyu Y-C, Skurtveit S, Verdoux H, Wang L-J, Yahni CZ, Bachmann CJ (2017) International trends in antipsychotic use: a study in 16 countries, 2005–2014. Eur Neuropsychopharmacol 27(10):1064–1076. 10.1016/j.euroneuro.2017.07.001 28755801 10.1016/j.euroneuro.2017.07.001

[CR46] Hojlund M, Pottegard A, Johnsen E, Kroken RA, Reutfors J, Munk-Jorgensen P, Correll, C.u. (2019) Trend in utilization and dosing of antipsychotic drugs in Scandinavia: comparison of 2006 and 2016. Br J Clin Pharmacol 85(7):1598–1606. 10.1111/bcp.13945 30927284 10.1111/bcp.13945 PMC6595354

[CR47] Institute for Health Metrics and Evaluation (IHME) (2024) GBD results. Interactive data visual. Seattle, WA: IHME, University of Washington. Last updated: May 2024. Last accessed: 22 Feb 2025. https://vizhub.healthdata.org/gbd-results/

[CR48] Italian Medicines Agency (2020) National report on medicines use in Italy. Year 2019. The Medicines Utilisation Monitoring Centre. Rome. ISBN: 979­12­80335­00­5. Last updated: 2020. Last accessed: 12 Feb 2025. https://www.aifa.gov.it/documents/20142/241052/OsMed_2019_Eng.pdf

[CR49] Italian Medicines Agency (2021) National report on medicines use in Italy. Year 2020. The Medicines Utilisation Monitoring Centre. Rome. ISBN: 979–12–80335–17–3. Last updated: 2021. Last accessed: 12 Feb 2025. https://www.aifa.gov.it/documents/20142/1542390/Rapporto-OsMed-2020_EN.pdf

[CR50] Italian Medicines Agency (2022) National report on medicines use in Italy. Year 2021. The Medicines Utilisation Monitoring Centre. Rome. ISBN: 979‐12‐80335‐26‐5. Last updated: 2022. Last accessed: 12 Feb 2025. https://www.aifa.gov.it/documents/20142/1740782/Rapporto-OsMed-2021_EN.pdf

[CR51] Italian Medicines Agency (2023) National report on medicines use in Italy. Year 2022. The Medicines Utilisation Monitoring Centre. Rome. ISBN: 979‐12‐80335‐31‐9. Last updated: 2023. Last accessed: 12 Feb 2025. https://www.aifa.gov.it/documents/20142/2143103/Rapporto-OsMed-2022_EN.pdf

[CR52] Italian Medicines Agency (2024) National report on medicines use in Italy. Year 2023. The Medicines Utilisation Monitoring Centre. Rome. ISBN: 979–12–80335–37–1. Last updated: 2024. Last accessed: 12 Feb 2025. https://www.aifa.gov.it/documents/20142/2594020/AIFA_Rapporto_OsMed_2023_EN.pdf

[CR53] Jaanson P (2007) Bipolaarse meeleoluhäirega patsientide ravijuhend [Guidelines for the treatment of patients with bipolar mood disorder]. Tartu Ülikooli Kliinikum. Last updated: 2007. Last accessed: 26 Feb 2025. https://www.kliinikum.ee/psyhhiaatriakliinik/lisad/ravi/ps-ravi/BPH_ravijuhis.pdf#:~:text=Eesti%20tingimustes%20on%20ehk%20k%C3%B5ige,ning%20nende%20v%C3%B5imaliku%20lisamis%20soodusravimite

[CR54] Jauhar S, Young AH (2019) Controversies in bipolar disorder; role of second-generation antipsychotic for maintenance therapy. Int J Bipolar Disord 7:10. 10.1186/s40345-019-0145-0 30915592 10.1186/s40345-019-0145-0 PMC6435763

[CR55] Jerrell JM, McIntyre RS, Black GB (2012) Economic grand rounds: economic costs of failure to monitor adverse effects of second-generation antipsychotics: an underestimated factor. Psychiatr Serv 63(3):202–204. 10.1176/appi.ps.20120p202 22388526 10.1176/appi.ps.20120p202

[CR56] Karanti A, Bobeck C, Osterman M, Kardell M, Tidemalm D, Runeson B, Lichtenstein P, Landén M (2015) Gender differences in the treatment of patients with bipolar disorder: a study of 7354 patients. J Affect Disord 174:303–309. 10.1016/j.jad.2014.11.058 25532077 10.1016/j.jad.2014.11.058

[CR57] Karanti A, Kardell M, Lundberg U, Landén M (2016) Changes in mood stabilizer prescription patterns in bipolar disorder. J Affect Disord 195:50–56. 10.1016/j.jad.2016.01.043 26859073 10.1016/j.jad.2016.01.043

[CR58] Kerner B (2014) Genetics of bipolar disorder. Appl Clin Genet 7:33–42. 10.2147/TACG.S39297 24683306 10.2147/TACG.S39297 PMC3966627

[CR59] Kessing LV (2019) Lithium as the drug of choice for maintenance treatment in bipolar disorder. Acta Psychiatr Scand 140:91–93. 10.1111/acps.13070 31310342 10.1111/acps.13070

[CR60] Kessing LV, Vradi E, Andersen PK (2016) Nationwide and population-based prescription patterns in bipolar disorder. Bipolar Disord 18(2):174–182. 10.1111/bdi.12371 26890465 10.1111/bdi.12371

[CR61] Koistinaho A, Poranen J, Tanskanen A, Tiihonen J, Taipale H, Lähteenvuo M (2023) Real-world use of pharamacological treatments for incident bipolar disorder: a Finnish nationwide cohort study. J Affect Disord 340:237–244. 10.1016/j.jad.2023.08.015 37557987 10.1016/j.jad.2023.08.015

[CR62] Kriner P, Brieger P, Pogarell O, Schüle C, Mußmann L, Korbmacher J, Seemüller M (2024) Treatment of bipolar depression: clinical practice vs. adherence to guidelines – data from a Bavarian drug surveillance project. Front Psychiatry 15. 10.3389/fpsyt.2024.1425549 10.3389/fpsyt.2024.1425549 PMC11250482 39015883

[CR63] Larsen JK, Porsdal V, Aarre TF, Koponen HJ, Aarnio J, Kleivenes OK, Board EA (2009) Mania in the Nordic countries: patients and treatment in the acute phase of the EMBLEM study. Nord J Psychiatry 63(4):285–291. 10.1080/08039480802663890 19140076 10.1080/08039480802663890

[CR64] Latvian State Agency of Medicines (2016) Baltic statistics on medicines 2013–2015. 2^nd^ edition. Riga, Latvia. ISBN 978–9934–8602–2–5. Last updated: 2016. Last accessed: 17 Feb 2025. https://www.zva.gov.lv/sites/default/files/2018-05/Baltic%20Statistics%20on%20Medicines%202013%20-%202015.pdf

[CR65] Li K, Richards E, Goes FS (2023) Racial differences in the major clinical symptom domains of bipolar disorder. Int J Bipolar Disord 11:17. 10.1186/s40345-023-00299-3 37166695 10.1186/s40345-023-00299-3 PMC10175527

[CR66] Lin Y, Mojtabai R, Goes FS, Zandi PP (2020) Trends in prescriptions of lithium and other medications for patients with bipolar disorder in office-based practices in the United States: 1996–2015. J Affect Disord 276:883–889. 10.1016/j.jad.2020.07.063 32739706 10.1016/j.jad.2020.07.063

[CR67] Ludwig W, Mühlbauer M, Seifert R (2021) Arzneiverordnungs-Report 2021. 1. (Berlin, Heidelberg: Springer). 10.1007/978-3-662-63825-5

[CR68] Ludwig W, Mühlbauer M, Seifert R (2023) Arzneiverordnungs-Report 2022. 1. (Berlin, Heidelberg: Springer). 10.1007/978-3-662-66303-5

[CR69] Ludwig W, Mühlbauer M, Seifert R (2024) Arzneiverordnungs-Report 2023. 1. (Berlin, Heidelberg: Springer). 10.1007/978-3-662-68371-2

[CR70] Ludwig W, Mühlbauer M, Seifert R (2025) Arzneiverordnungs-Report 2024. 1. (Berlin, Heidelberg: Springer). 10.1007/978-3-662-70594-0

[CR71] Lumezi L (2023) Primjena litija u psihijatriji. Master’s thesis. University of Zagreb, School of Medicine. Last updated: 2023. Last accessed: 26 Feb 2025. https://urn.nsk.hr/urn:nbn:hr:105:435661

[CR72] Magioncalda P, Martino M (2022) A unified model of the pathophysiology of bipolar disorder. Mol Psychiatry 27:202–211. 10.1038/s41380-021-01091-4 33859358 10.1038/s41380-021-01091-4

[CR73] Malhi GS, Bauer M (2023) Lithium first: not merely first line. Bipolar Disord 25(1):7–8. 10.1111/bdi.13299 36808784 10.1111/bdi.13299

[CR74] Malhi GS, Tanious M (2011) Optimal frequency of lithium administration in the treatment of bipolar disorder. CNS Drugs 25:289–298. 10.2165/11586970-000000000-00000 21425882 10.2165/11586970-000000000-00000

[CR75] Malhi GS, Gessler D, Outhred T (2017) The use of lithium for the treatment of bipolar disorder: recommendations from clinical practice guidelines. J Affect Disord 217:266–280. 10.1016/j.jad.2017.03.052 28437764 10.1016/j.jad.2017.03.052

[CR76] Malhi GS, Bell E, Jadidi M, Gitlin M, Bauer M (2023) Countering the declining use of lithium therapy: a call to arms. International Journal of Bipolar Disorders 11(1):30. 10.1186/s40345-023-00310-x 37633877 10.1186/s40345-023-00310-x PMC10460327

[CR77] McKinney W (2010) Data structures for statistical computing in Python. Proceedings of the 9th Python in Science Conference 51–56. Last Accessed: 30 Jan 2025. https://pub.curvenote.com/01908378-3686-7168-a380-d82bbf21c799/public/mckinney-57fc0d4e8a08cd7f26a4b8bf468a71f4.pdf

[CR78] McKnight RF, Adida M, Budge K, Stockton S, Goodwin GM, Geddes JR (2012) Lithium toxicity profile: a systematic review and meta-analysis. Lancet (London, England) 379(9817):721–728. 10.1016/S0140-6736(11)61516-X 22265699 10.1016/S0140-6736(11)61516-X

[CR79] Merikangas KR, Jin R, He J et al (2011) Prevalence and correlates of bipolar spectrum disorder in the World Mental Health Survey Initiative. Arch Gen Psychiatry 68(3):241–251. 10.1001/archgenpsychiatry.2011.12 21383262 10.1001/archgenpsychiatry.2011.12 PMC3486639

[CR80] Ministerio de Sanidad (2020) Guía de Práctica Clínica de Prevención y Tratamiento de la Cunducta Suicida. Last updated : September 2020. Last accessed: 04 March 2025. 10.46995/gpc_481. https://portal.guiasalud.es/gpc/conducta-suicida/

[CR81] Ministerio de Sanidad (2023) Guía de Práctica Clínica sobre el Manejo de la Depresión en el Adulto. Last updated: May 2023. Last accessed: 04 March2025. 10.46995/gpc_534. https://portal.guiasalud.es/gpc/depresion-adulto/

[CR82] Ministerio de Sanidad (2025) Consumo de Productos Farmacéuticos. Datos de consumo de recetas médicas del SNS según clasificación Anatómica-Terapéutica-Química (ATC). Last updated: 2025. Last accessed: 13 Feb 2025. https://www.sanidad.gob.es/areas/farmacia/consumoMedicamentos/ATC/home.htm

[CR83] O’Connell KS, Coombes BJ (2021) Genetic contributions to bipolar disorder: current status and future directions. Psychol Med 51(13):2156–2167. 10.1017/S0033291721001252 33879273 10.1017/S0033291721001252 PMC8477227

[CR84] Oliva V, Fico G, De Prisco M, Gonda X, Rosa AR, Vieta E (2025) Bipolar disorders: an update on critical aspects. Lancet Regional Health – Europe 48:101135. 10.1016/j.lanepe.2024.101135 10.1016/j.lanepe.2024.101135 PMC11732062 39811787

[CR85] Ortiz-Orendain J, Castiello-de Obeso S, Colunga-Lozano LE, Hu Y, Maayan N, and Adams CE (2017) Antipsychotic combinations for schizophrenia. Cochrane Database Syst Rev 6(6):CD009005. 10.1002/14651858.CD009005.pub2 10.1002/14651858.CD009005.pub2 PMC6481822 28658515

[CR86] Pacciardi B, Palagini L, Mainardi C, Cotugno B, Cargioli C, Perugi G, Di Fiorino M (2017) Attitude toward prescription and clinical monitoring of lithium salts in a sample of Italian psychiatrists: preliminary data. J Psychopathol 4(23):172–179. Last updated: 2017. Last accessed: 26 Feb 2025. http://www.jpsychopathol.it/wp-content/uploads/2018/02/06_Pacciardi-1.pdf

[CR87] Pérez de Mendiola X, Hidalgo-Mazzei D, Vieta E, González-Pinto A (2021) Overview of lithium’s use: a nationwide survey. International Journal of Bipolar Disorders 9(1):10. 10.1186/s40345-020-00215-z 33687600 10.1186/s40345-020-00215-z PMC7941362

[CR88] Puglisi-Allegra S, Ruggieri S, Fornai F (2021) Translational evidence for lithium-induced brain plasticity and neuroprotection in the treatment of neuropsychiatric disorders. Transl Psychiatry 11:366. 10.1038/s41398-021-01492-7 34226487 10.1038/s41398-021-01492-7 PMC8257731

[CR89] Python Package Index (pypi) (2024) openpyxl 3.1.5. pip install openpyxl. Last updated: Juny 2024. Last Accessed: 30 Jan 2025. https://pypi.org/project/openpyxl/#description

[CR90] Renes JW, Regeer EJ, van der Voort TY et al (2014) Treatment of bipolar disorder in the Netherlands and concordance with treatment guidelines: study protocol of an observational, longitudinal study on naturalistic treatment of bipolar disorder in everyday clinical practice. BMC Psychiatry 14:58. 10.1186/1471-244X-14-58 24576061 10.1186/1471-244X-14-58 PMC3940255

[CR91] Renes JW, Regeer EJ, Hoogendoorn AW, Nolen WA, Kupka RW (2018) A nationwide study on concordance with multimodal treatment guidelines in bipolar disorder. International Journal of Bipolar Disorders 6(1):22. 10.1186/s40345-018-0130-z 30341458 10.1186/s40345-018-0130-z PMC6195496

[CR92] Rotar AM, van den Berg MJ, Klazinga NS (2020) An expert-based mapping of healthcare system strategies to support rational drug prescribing in primary care across 13 European countries. Health Res Policy Sys 18:102. 10.1186/s12961-020-00605-w 10.1186/s12961-020-00605-w PMC7493959 32933555

[CR93] Rote Liste (2025) Quilonum retard Retardtabletten. Fachinformation. Last updated: January 2025. Last accessed: 28 March 2025. https://www.rote-liste.de/suche/praep/5849- 0/Quilonum%C2%AE%20retard%20Retardtabletten

[CR94] Rowland TA, Marwaha S (2018) Epidemiology and risk factors for bipolar disorder. Therapeutic Advances in Psychopharmacology 8(9):251–269. 10.1177/2045125318769235 30181867 10.1177/2045125318769235 PMC6116765

[CR95] Rybakowski JK, Suwalska A, Hajek T (2018) Clinical perspectives of lithium’s neuroprotective effect. Pharmacopsychiatry 51(5):194–199. 10.1055/s-0043-124436 29270949 10.1055/s-0043-124436

[CR96] Samalin L, Vieta E, Okasha T et al (2016) Management of bipolar disorder in the intercontinental region: an international, multicenter, non-interventional, cross-sectional study in real-life conditions. Sci Rep 6:25920. 10.1038/srep25920 27181262 10.1038/srep25920 PMC4867470

[CR97] Schwabe U and Ludwig W (2020) Arzneiverordnungs-Report 2020. 1. (Berlin, Heidelberg: Springer). 10.1007/978-3-662-62168-4

[CR98] Schwabe U and Paffrath D (2014) Arzneiverordnungs-Report 2014. 1. (Berlin, Heidelberg: Springer). 10.1007/978-3-662-43487-1

[CR99] Schwabe U and Paffrath D (2015) Arzneiverordnungs-Report 2015. 1. (Berlin, Heidelberg: Springer). 10.1007/978-3-662-47186-9

[CR100] Schwabe U and Paffrath D (2016) Arzneiverordnungs-Report 2016. 1. (Berlin, Heidelberg: Springer). 10.1007/978-3-662-50351-5

[CR101] Schwabe U and Paffrath D (2017) Arzneiverordnungs-Report 2017. 1. (Berlin, Heidelberg: Springer). 10.1007/978-3-662-54630-7

[CR102] Schwabe U, Paffrath D, Ludwig W, and Klauber J (2018) Arzneiverordnungs-Report 2018. 1. (Berlin, Heidelberg: Springer). 10.1007/978-3-662-57386-0

[CR103] Schwabe U, Paffrath D, Ludwig W, and Klauber J (2019) Arzneiverordnungs-Report 2019. 1. (Berlin, Heidelberg: Springer). 10.1007/978-3-662-59046-1

[CR104] Seabold S, Perktold J (2010) Statsmodels: econometric and statistical modeling with Python. Proceedings of the 9th Python in Science Conference (SciPy 2010), Austin, TX, 28 June–3 July 2010, pp. 92–96. Last Accessed: 30 Jan 2025. https://pub.curvenote.com/01908379-2a1a-7a74-a157-6a0df64b92f2/public/seabold-34d6671a7bae7c2c09a284f57c0422d9.pdf

[CR105] Shine B, McKnight RF, Leaver L, Geddes JR (2015) Long-term effects of lithium on renal, thyroid, and parathyroid function: a retrospective analysis of laboratory data. Lancet (London, England) 386(9992):461–468. 10.1016/S0140-6736(14)61842-0 26003379 10.1016/S0140-6736(14)61842-0

[CR106] Shorter E (2009) The history of lithium therapy. Bipolar disorders, 11 Suppl 2(Suppl 2):4–9. 10.1111/j.1399-5618.2009.00706.x 10.1111/j.1399-5618.2009.00706.x PMC3712976 19538681

[CR107] Shuy YK, Santharan S, Chew QH, Sim K (2024) International trends in lithium use for pharmacotherapy and clinical correlates in bipolar disorder: a scoping review. Brain Sci 14(1):102. 10.3390/brainsci14010102 38275522 10.3390/brainsci14010102 PMC10813799

[CR108] Sköld M, Rolstad S, Joas E, Kardell M, Pålsson E, Goodwin GM, Landén M (2021) Regional lithium prescription rates and recurrence in bipolar disorder. International Journal of Bipolar Disorders 9(1):18. 10.1186/s40345-021-00223-7 34061259 10.1186/s40345-021-00223-7 PMC8167923

[CR109] Socialstyrelsen (2025) Statistikdatabas för läkemedel. Last updated: January 2025. Last accessed: 18 Feb 2025. https://sdb.socialstyrelsen.se/if_lak/val.aspx

[CR110] Socialstyrelsen (2021) Nationella riktlinjer för vård vid depression och ångestsyndrom. Stöd för styrning och ledning. ISBN 978–91–7555–563–8. Last updated: April 2021. Last accessed: 4 March 2025. https://www.socialstyrelsen.se/globalassets/sharepoint-dokument/artikelkatalog/nationella-riktlinjer/2021-4-7339.pdf

[CR111] State Medicines Control Agency of Lithuania (2019) Baltic statistics on medicines 2016–2018. Vilnius, Lithuania. ISBN 978–609–462–139–0. Last updated: 2019. Last accessed: 17 Feb 2025. https://www.zva.gov.lv/sites/default/files/2020-01/Baltic%20statistics_3rd%20edition.pdf

[CR112] Sundhedsdatastyrelsen (2024) Medstat.dk. The Danish Health Data Authority, Copenhagen. Last updated: March 2024. Last accessed: 27 Feb 2025. https://www.medstat.dk/en

[CR113] Strejilevich SA, Urtueta-Baamonde M, Teitelbaum J, Martino DJ, Marengo E, Igoa A, Fassi G, Cetkovich-Bakmas M (2011) Conceptos clínicos asociados a la subestimación del litio en el tratamiento del Trastorno Bipolar [Clinical concepts associated with lithium underutilization in the treatment of bipolar disorder]. Vertex (Buenos Aires, Argentina) 22(Suppl):3–20 21898968

[CR114] Stroup TS, Gray N (2018) Management of common adverse effects of antipsychotic medications. World Psychiatry (WPA) 17(3):341–356. 10.1002/wps.20567 30192094 10.1002/wps.20567 PMC6127750

[CR115] Tian F, Chen Z, Zeng Y, Feng Q, Chen X (2023) Prevalence of use of potentially inappropriate medications among older adults worldwide: a systematic review and meta-analysis. JAMA Netw Open 6(8):e2326910. 10.1001/jamanetworkopen.2023.26910 37531105 10.1001/jamanetworkopen.2023.26910 PMC10398411

[CR116] Uçok A, Gaebel W (2008) Side effects of atypical antipsychotics: a brief overview. World Psychiatry : Official Journal of the World Psychiatric Association (WPA) 7(1):58–62. 10.1002/j.2051-5545.2008.tb00154.x 18458771 10.1002/j.2051-5545.2008.tb00154.x PMC2327229

[CR117] Vieta E, Langosch JM, Figueira ML, Blasco-Colmenares E, Medina E, Morena-Manzanaro M, Ganzalez MA, Bellivier F (2013) Clinical management and burden of bipolar disorder: results from a multinational longitudinal study (WAVE-bd). Int J Neuropsychopharmacol 16(8):1719–1732. 10.1017/S1461145713000278 10.1017/S1461145713000278 23663490

[CR118] World Health Organization (WHO) (2012) Combination of two or more antipsychotic medications for psychotic disorders. Last updated: June 2012. Last accessed: 25 Feb 2025. https://cdn.who.int/media/docs/default-source/mental-health/mhgap/psychosis-and-bipolar-disorders/combination-of-two-or-more-antipsychotic-medications-for-psychotic-disorders.pdf?sfvrsn=39f68d81_0

[CR119] World Health Organization (WHO) (2022) New WHO report: Europe can reverse its obesity “epidemic”. Newsroom. Last updated: May 2022. Last accessed: 23 Feb 2025. https://www.who.int/europe/news-room/03-05-2022-new-who-report--europe-can-reverse-its-obesity--epidemic

[CR120] World Health Organization (WHO) (2024a) ATC/DDD Index 2025. WHO Collaborating Centre for Drug Statistics Methodology. Oslo, Norway. Last updated: December 2024. Last accessed: 28 Feb 2025. https://atcddd.fhi.no/atc_ddd_index/

[CR121] World Health Organization (WHO) (2024b) Obesity and overweight. Fact sheet. Last updated: March 2024. Last accessed: 23 Feb 2025. https://www.who.int/news-room/fact-sheets/detail/obesity-and-overweight

[CR122] World Health Organization (WHO) (2025) Defined Daily Dose (DDD). Definition and general considerations. ATC-DDD Toolkit. Last updated: 2025. Last accessed: 25 May 2025. https://www.who.int/tools/atc-ddd-toolkit/about-ddd

[CR123] Zhao Z, Okusaga OO, Quevedo J, Soares JC, Teixeira AL (2016) The potential association between obesity and bipolar disorder: a meta-analysis. Journal of Affective Disorders. Volume 202, 15 September 2016, pages 120–123. 10.1016/j.jad.2016.05.059 10.1016/j.jad.2016.05.059 27262632

