# Supplementary material for: Evidence of lithium underuse in bipolar disorder: analysis of lithium and antipsychotic consumption, prediction of future trends, regional disparities and indicators of rational and inappropriate use in Europe
Source: Naunyn Schmiedebergs Arch Pharmacol. 2025 Jun 28;398(12):18049–70. doi: 10.1007/s00210-025-04389-0 (PMC12678625; doi:10.1007/s00210-025-04389-0)
Supplement: Supplementary file 1 — (DOCX 53.0 KB) [file 210_2025_4389_MOESM1_ESM.docx]

**Evidence of lithium underuse in bipolar disorder: Analysis of lithium and antipsychotic consumption, prediction of future trends, regional disparities and indicators of rational and inappropriate use in Europe**

**Lilly Josephine Bindel and Roland Seifert**

Supplemental Tables

***Table S1:*** *Consumption data in DID prescriptions for lithium (ATC N05AN). For each country, the covered years are depicted. “*” means that the consumption was too low to be further quantified.*

| **year** | **Croatia** | **Denmark** | **Estonia** | **Finland** | **Germany** | **Iceland** | **Italy** | **Netherlands** | **Norway** | **Spain** | **Sweden** |
| --- | --- | --- | --- | --- | --- | --- | --- | --- | --- | --- | --- |
| **1997** |  | 1.2 |  |  |  |  |  |  |  |  |  |
| **1998** |  | 1.2 |  |  |  |  |  |  |  |  |  |
| **1999** |  | 1.1 |  |  |  |  |  |  |  |  |  |
| **2000** |  | 1.2 |  |  |  |  |  |  |  |  |  |
| **2001** |  | 1.2 |  |  |  |  |  |  |  |  |  |
| **2002** |  | 1.2 |  |  |  |  |  |  |  |  |  |
| **2003** |  | 1.1 |  |  |  |  |  |  |  |  |  |
| **2004** |  | 1.1 |  |  |  |  |  |  | 1.69 |  |  |
| **2005** |  | 1.1 |  |  |  |  |  |  | 1.70 |  |  |
| **2006** |  | 1.1 |  |  |  |  |  |  | 1.66 |  | 1.50 |
| **2007** |  | 1.1 |  |  |  |  |  |  | 1.64 |  | 1.55 |
| **2008** |  | 1.1 |  |  |  |  |  |  | 1.66 |  | 1.55 |
| **2009** |  | 1.1 |  |  |  |  |  |  | 1.66 |  | 1.65 |
| **2010** |  | 1.1 | 0.16 |  |  |  |  |  | 1.61 | 0.90 | 1.60 |
| **2011** |  | 1.1 | 0.16 |  |  |  |  |  | 1.56 | 0.89 | 1.60 |
| **2012** |  | 1.1 | 0.17 |  |  |  |  |  | 1.55 | 0.87 | 1.60 |
| **2013** |  | 1.1 | 0.17 |  | 0.71 |  |  |  | 1.51 | 0.87 | 1.65 |
| **2014** |  | 1.1 | 0.19 |  | 0.73 |  | 0.30 |  | 1.47 | 0.88 | 1.60 |
| **2015** |  | 1.1 | 0.19 |  | 0.72 | 1.40 | 0.40 |  | 1.46 | 0.88 | 1.60 |
| **2016** |  | 1.1 | 0.20 |  | 0.71 | 1.40 | 0.40 |  | 1.44 | 0.89 | 1.60 |
| **2017** |  | 1.1 | 0.21 | 0.94 | 0.71 | 1.30 | 0.40 |  | 1.44 | 0.47 | 1.60 |
| **2018** |  | 1.1 | 0.23 | 0.97 | 0.70 | 1.30 | 0.30 |  | 1.44 | 0.88 | 1.60 |
| **2019** | 0.00* | 1.1 | 0.36 | 0.98 | 0.74 | 1.30 | 0.40 | 1.15 | 1.46 | 0.89 | 1.60 |
| **2020** | 0.00* | 1.1 | 0.36 | 0.99 | 0.76 | 1.20 | 0.40 | 1.26 | 1.45 | 0.89 | 1.60 |
| **2021** | 0.28 | 1.1 | 0.34 |  | 0.75 | 1.10 | 0.40 | 1.23 |  | 0.88 | 1.60 |
| **2022** | 0.26 | 1.1 | 0.29 |  | 0.74 | 1.10 | 0.40 | 1.20 |  | 0.88 | 1.60 |
| **2023** |  | 1.1 | 0.26 |  |  | 1.20 | 0.40 | 1.17 |  | 0.86 | 1.60 |
| **2024** |  |  |  |  |  | 1.20 |  |  |  |  |  |

***Table S2:*** *Consumption data in DID prescriptions for antipsychotics (ATC N05A). For each country, the covered years are depicted. “*” means that the consumption was too low to be further quantified.*

| **year** | **Croatia** | **Denmark** | **Estonia** | **Finland** | **Germany** | **Iceland** | **Italy** | **Netherlands** | **Norway** | **Spain** | **Sweden** |
| --- | --- | --- | --- | --- | --- | --- | --- | --- | --- | --- | --- |
| **1997** |  | 9.10 |  |  |  |  |  |  |  |  |  |
| **1998** |  | 9.50 |  |  |  |  |  |  |  |  |  |
| **1999** |  | 9.70 |  |  |  |  |  |  |  |  |  |
| **2000** |  | 10.10 |  |  |  |  |  |  |  |  |  |
| **2001** |  | 10.70 |  |  |  |  |  |  |  |  |  |
| **2002** |  | 11.30 |  |  |  |  |  |  |  |  |  |
| **2003** |  | 12.00 |  |  |  |  |  |  |  |  |  |
| **2004** |  | 12.50 |  |  |  |  |  |  | 22.78 |  |  |
| **2005** |  | 13.00 |  |  |  |  |  |  | 22.97 |  |  |
| **2006** |  | 12.90 |  |  |  |  |  |  | 22.80 |  | 8.50 |
| **2007** | 8.01 | 12.90 |  |  |  |  |  |  | 22.46 |  | 8.35 |
| **2008** | 8.26 | 13.20 |  |  |  |  |  |  | 21.83 |  | 8.50 |
| **2009** | 10.09 | 13.50 |  |  |  |  |  |  | 21.55 |  | 8.55 |
| **2010** | 11.13 | 13.90 | 6.10 |  |  |  |  |  | 21.29 | 10.87 | 8.85 |
| **2011** | 11.82 | 14.30 | 6.38 |  |  |  |  |  | 21.07 | 11.25 | 8.95 |
| **2012** | 12.21 | 14.70 | 6.93 |  |  |  |  |  | 21.11 | 11.30 | 9.05 |
| **2013** | 12.78 | 14.30 | 7.58 |  | 10.99 |  |  |  | 20.95 | 11.55 | 9.20 |
| **2014** | 13.21 | 14.10 | 8.27 |  | 11.60 |  | 8.40 |  | 21.22 | 11.86 | 9.25 |
| **2015** | 13.95 | 13.90 | 8.66 |  | 11.24 | 12.20 | 8.90 |  | 21.83 | 12.24 | 9.45 |
| **2016** | 14.28 | 13.70 | 9.16 |  | 11.46 | 11.90 | 9.10 |  | 22.66 | 12.53 | 9.55 |
| **2017** | 13.74 | 13.60 | 8.99 | 20.60 | 11.41 | 11.70 | 9.30 |  | 23.58 | 12.78 | 9.55 |
| **2018** | 14.32 | 13.60 | 8.67 | 21.12 | 11.39 | 11.60 | 9.50 |  | 24.33 | 12.96 | 9.55 |
| **2019** | 15.14 | 13.50 | 9.58 | 21.38 | 12.13 | 11.80 | 9.70 | 8.10 | 24.54 | 13.14 | 9.60 |
| **2020** | 15.35 | 13.60 | 10.05 | 22.15 | 12.62 | 12.50 | 10.10 | 8.23 | 25.03 | 13.54 | 9.75 |
| **2021** | 15.81 | 13.60 | 10.18 |  | 12.70 | 12.70 | 10.10 | 8.23 |  | 13.95 | 9.75 |
| **2022** | 16.05 | 13.60 | 10.71 |  | 12.37 | 12.00 | 10.40 | 8.22 |  | 14.31 | 9.80 |
| **2023** |  | 13.60 | 10.80 |  |  | 13.30 | 10.60 | 8.19 |  | 14.50 | 9.85 |
| **2024** |  |  |  |  |  | 14.00 |  |  |  |  |  |

***Table S3:*** *Lithium treatment coverage for bipolar disorder. For each country, coverage is calculated using its DID prescriptions (see Table S1) and the prevalence of the corresponding year (IHME 2024). As no prevalence data were provided after 2021, the prevalence of 2021 was used to calculate the coverage for the years 2022-2024.*

| **year** | **Croatia** | **Denmark** | **Estonia** | **Finland** | **Germany** | **Iceland** | **Italy** | **Netherlands** | **Norway** | **Spain** | **Sweden** | **prevalence of bipolar disorder** |
| --- | --- | --- | --- | --- | --- | --- | --- | --- | --- | --- | --- | --- |
| **1997** |  | 26.7% |  |  |  |  |  |  |  |  |  | 0.45% |
| **1998** |  | 26.7% |  |  |  |  |  |  |  |  |  | 0.45% |
| **1999** |  | 23.9% |  |  |  |  |  |  |  |  |  | 0.46% |
| **2000** |  | 26.1% |  |  |  |  |  |  |  |  |  | 0.46% |
| **2001** |  | 26.1% |  |  |  |  |  |  |  |  |  | 0.46% |
| **2002** |  | 26.1% |  |  |  |  |  |  |  |  |  | 0.46% |
| **2003** |  | 23.4% |  |  |  |  |  |  |  |  |  | 0.47% |
| **2004** |  | 23.4% |  |  |  |  |  |  | 35.9% |  |  | 0.47% |
| **2005** |  | 23.4% |  |  |  |  |  |  | 36.1% |  |  | 0.47% |
| **2006** |  | 23.4% |  |  |  |  |  |  | 35.4% |  | 31.9% | 0.47% |
| **2007** |  | 23.4% |  |  |  |  |  |  | 34.9% |  | 33.0% | 0.47% |
| **2008** |  | 22.9% |  |  |  |  |  |  | 34.6% |  | 32.3% | 0.48% |
| **2009** |  | 22.9% |  |  |  |  |  |  | 34.5% |  | 34.4% | 0.48% |
| **2010** |  | 22.9% | 3.3% |  |  |  |  |  | 33.6% | 18.8% | 33.4% | 0.48% |
| **2011** |  | 22.9% | 3.3% |  |  |  |  |  | 32.5% | 18.5% | 33.3% | 0.48% |
| **2012** |  | 22.9% | 3.5% |  |  |  |  |  | 32.3% | 18.1% | 33.3% | 0.48% |
| **2013** |  | 22.9% | 3.5% |  | 14.9% |  |  |  | 31.5% | 18.1% | 34.4% | 0.48% |
| **2014** |  | 22.9% | 4.0% |  | 15.2% |  | 6.3% |  | 30.6% | 18.3% | 33.3% | 0.48% |
| **2015** |  | 22.4% | 3.9% |  | 14.7% | 28.6% | 8.2% |  | 29.8% | 18.0% | 32.7% | 0.49% |
| **2016** |  | 22.4% | 4.1% |  | 14.5% | 28.6% | 8.2% |  | 29.5% | 18.2% | 32.6% | 0.49% |
| **2017** |  | 22.4% | 4.3% | 19.2% | 14.4% | 26.5% | 8.2% |  | 29.5% | 9.6% | 32.6% | 0.49% |
| **2018** |  | 22.4% | 4.7% | 19.8% | 14.3% | 26.5% | 6.1% |  | 29.3% | 18.0% | 32.6% | 0.49% |
| **2019** |  | 22.4% | 7.3% | 20.0% | 15.0% | 26.5% | 8.2% | 23.5% | 29.7% | 18.2% | 32.6% | 0.49% |
| **2020** |  | 22.4% | 7.3% | 20.2% | 15.5% | 24.5% | 8.2% | 25.7% | 29.5% | 18.2% | 32.6% | 0.49% |
| **2021** | 5.7% | 22.4% | 6.9% |  | 15.3% | 22.4% | 8.2% | 25.0% |  | 18.0% | 32.6% | 0.49% |
| **2022** | 5.3% | 22.4% | 5.9% |  | 15.2% | 22.4% | 8.2% | 24.5% |  | 18.0% | 32.6% |  |
| **2023** |  | 22.4% | 5.3% |  |  | 24.5% | 8.2% | 24.0% |  | 17.6% | 32.6% |  |
| **2024** |  |  |  |  |  | 24.5% |  |  |  |  |  |  |

***Table S4:*** *Ratio of DID prescriptions of antipsychotics exclusive lithium versus lithium for analysed countries.*

| **year** | **Croatia** | **Denmark** | **Estonia** | **Finland** | **Germany** | **Iceland** | **Italy** | **Netherlands** | **Norway** | **Spain** | **Sweden** |
| --- | --- | --- | --- | --- | --- | --- | --- | --- | --- | --- | --- |
| **1997** |  | 6.58 |  |  |  |  |  |  |  |  |  |
| **1998** |  | 6.92 |  |  |  |  |  |  |  |  |  |
| **1999** |  | 7.82 |  |  |  |  |  |  |  |  |  |
| **2000** |  | 7.42 |  |  |  |  |  |  |  |  |  |
| **2001** |  | 7.92 |  |  |  |  |  |  |  |  |  |
| **2002** |  | 8.42 |  |  |  |  |  |  |  |  |  |
| **2003** |  | 9.91 |  |  |  |  |  |  |  |  |  |
| **2004** |  | 10.36 |  |  |  |  |  |  | 12.50 |  |  |
| **2005** |  | 10.82 |  |  |  |  |  |  | 12.54 |  |  |
| **2006** |  | 10.73 |  |  |  |  |  |  | 12.72 |  | 4.66 |
| **2007** |  | 10.73 |  |  |  |  |  |  | 12.71 |  | 4.38 |
| **2008** |  | 11.00 |  |  |  |  |  |  | 12.13 |  | 4.48 |
| **2009** |  | 11.27 |  |  |  |  |  |  | 12.02 |  | 4.18 |
| **2010** |  | 11.64 | 37.13 |  |  |  |  |  | 12.21 | 11.08 | 4.53 |
| **2011** |  | 12.00 | 38.88 |  |  |  |  |  | 12.51 | 11.64 | 4.60 |
| **2012** |  | 12.36 | 39.76 |  | 14.38 |  |  |  | 12.60 | 11.99 | 4.66 |
| **2013** |  | 12.00 | 43.59 |  | 14.90 |  |  |  | 12.86 | 12.28 | 4.58 |
| **2014** |  | 11.82 | 42.53 |  | 14.64 |  | 27.00 |  | 13.43 | 12.48 | 4.78 |
| **2015** |  | 11.64 | 44.58 |  | 15.13 | 7.71 | 21.25 |  | 13.95 | 12.91 | 4.91 |
| **2016** |  | 11.45 | 44.80 |  | 15.14 | 7.50 | 21.75 |  | 14.68 | 13.08 | 4.97 |
| **2017** |  | 11.36 | 41.81 | 20.91 | 15.24 | 8.00 | 22.25 |  | 15.32 | 26.19 | 4.97 |
| **2018** |  | 11.36 | 36.70 | 20.77 | 15.49 | 7.92 | 30.67 |  | 15.93 | 13.73 | 4.97 |
| **2019** |  | 11.27 | 25.61 | 20.82 | 15.59 | 8.08 | 23.25 | 6.04 | 15.87 | 13.76 | 5.00 |
| **2020** |  | 11.36 | 26.92 | 21.37 | 15.91 | 9.42 | 24.25 | 5.53 | 16.30 | 14.21 | 5.10 |
| **2021** | 55.46 | 11.36 | 28.94 |  | 15.63 | 10.55 | 24.25 | 5.72 |  | 14.85 | 5.10 |
| **2022** | 60.73 | 11.36 | 35.93 |  |  | 9.91 | 25.00 | 5.85 |  | 15.26 | 5.13 |
| **2023** |  | 11.36 | 40.54 |  |  | 10.08 | 25.50 | 5.98 |  | 15.86 | 5.16 |
| **2024** |  |  |  |  |  | 10.67 |  |  |  |  |  |

***Table S5:*** *Python code and output for ADF test and estimation of generalised optimal parameters for all countries.*

| !pip install pandas pmdarima openpyxl statsmodels |
| --- |
|  |
| import pandas as pd |
| from pmdarima import auto_arima |
| from statsmodels.tsa.stattools import adfuller |
| from google.colab import files |
|  |
| # Step 1: Upload the file |
| print("Please upload your Excel file:") |
| uploaded = files.upload() |
|  |
| # Check the name of the uploaded file |
| file_path = list(uploaded.keys())[0] |
| print(f"File '{file_path}' successfully uploaded.") |
|  |
| # Step 2: Load the Excel file into a DataFrame |
| df = pd.read_excel(file_path, sheet_name=0) # Load the first sheet |
|  |
| # Step 3: Verify the "Time period" column and set as index |
| if 'Time period' in df.columns: |
| print("'Time period' column found. Here are the first few values:") |
| print(df['Time period'].head()) |
| df.set_index('Time period', inplace=True) |
| else: |
| raise ValueError("The column 'Time period' is missing. Please check your file.") |
|  |
| # Step 4: Calculate the mean of values for each time period |
| all_series = df.mean(axis=1) # Calculate the mean across columns |
|  |
| # Step 5: Perform ADF test and determine differencing order (up to d=2) |
| try: |
| adf_result = adfuller(all_series.dropna()) |
| p_value = adf_result[1] |
|  |
| if p_value > 0.05: |
| diff_series = all_series.diff().dropna() |
| if len(diff_series) > 1: |
| try: |
| adf_result_diff1 = adfuller(diff_series) |
| p_value_diff1 = adf_result_diff1[1] |
| if p_value_diff1 > 0.05: |
| diff_series2 = diff_series.diff().dropna() |
| if len(diff_series2) > 1: |
| d = 2 |
| else: |
| d = 1 |
| else: |
| d = 1 |
| except ValueError: |
| d = 0 |
| else: |
| d = 0 |
| else: |
| d = 0 |
| except ValueError: |
| d = 0 |
|  |
| # Step 6: Find optimal ARIMA parameters using BIC |
| model = auto_arima( |
| all_series.dropna(), |
| seasonal=False, |
| stepwise=True, |
| suppress_warnings=True, |
| trace=True, # You might want to set this to False for cleaner output |
| error_action='ignore', |
| information_criterion='bic', |
| d=d, |
| max_d=2 |
| ) |
|  |
| # Print the best model parameters |
| print("Best model:", model) |
|  |
| # Extract and save the parameters |
| p, d, q = model.order |
| with open("global_arima_parameters.txt", "w") as f: |
| f.write(f"p: {p}\n") |
| f.write(f"d: {d}\n") |
| f.write(f"q: {q}\n") |
|  |
| print("Global ARIMA parameters saved to global_arima_parameters.txt") |

**Output**

**Best model lithium: ARIMA(0,1,0)(0,0,0)[0] Total fit time: 0.649 seconds**

**Best model antipsychotics: ARIMA(0,1,0)(0,0,0)[0] Total fit time: 0.397 seconds**

***Table S6:*** *Forecast of DID prescriptions for lithium (ATC N05AN) until 2030.*

| **year** | **2021** | **2022** | **2023** | **2024** | **2025** | **2026** | **2027** | **2028** | **2029** | **2030** |
| --- | --- | --- | --- | --- | --- | --- | --- | --- | --- | --- |
| **Denmark** | . | . | . | 1.1 | 1.09 | 1.09 | 1.08 | 1.08 | 1.08 | 1.07 |
| **Estonia** | . | . | . | 0.27 | 0.28 | 0.28 | 0.29 | 0.3 | 0.31 | 0.31 |
| **Finland** | 1.01 | 1.02 | 1.04 | 1.06 | 1.07 | 1.09 | 1.11 | 1.12 | 1.14 | 1.16 |
| **Germany** | . | . | 0.75 | 0.75 | 0.75 | 0.76 | 0.76 | 0.76 | 0.77 | 0.77 |
| **Iceland** | . | . | . | . | 1.18 | 1.16 | 1.13 | 1.11 | 1.09 | 1.07 |
| **Italy** | . | . | . | 0.41 | 0.42 | 0.43 | 0.44 | 0.46 | 0.47 | 0.48 |
| **Netherlands** | . | . | . | 1.18 | 1.19 | 1.19 | 1.2 | 1.2 | 1.21 | 1.21 |
| **Norway** | 1.43 | 1.42 | 1.4 | 1.39 | 1.37 | 1.36 | 1.34 | 1.33 | 1.31 | 1.3 |
| **Spain** | . | . | . | 0.86 | 0.85 | 0.85 | 0.85 | 0.84 | 0.84 | 0.84 |
| **Sweden** | . | . | . | 1.6 | 1.61 | 1.62 | 1.62 | 1.63 | 1.63 | 1.64 |
| For each model, forecasts start after the last non-missing in the range of the requested estimation period and end at the last period for which non-missing values of all the predictors are available or at the end date of the requested forecast period, whichever is earlier. | | | | | | | | | | |

***Table S7:*** *Forecast of DID prescriptions for antipsychotics (ATC N05A) until 2030.*

| **year** | **2021** | **2022** | **2023** | **2024** | **2025** | **2026** | **2027** | **2028** | **2029** | **2030** |
| --- | --- | --- | --- | --- | --- | --- | --- | --- | --- | --- |
| **Croatia** | . | . | 16.59 | 17.12 | 17.66 | 18.19 | 18.73 | 19.27 | 19.8 | 20.34 |
| **Denmark** | . | . | . | 13.77 | 13.95 | 14.12 | 14.29 | 14.47 | 14.64 | 14.81 |
| **Estonia** | . | . | . | 11.16 | 11.52 | 11.88 | 12.25 | 12.61 | 12.97 | 13.33 |
| **Finland** | 22.67 | 23.18 | 23.7 | 24.22 | 24.73 | 25.25 | 25.77 | 26.28 | 26.8 | 27.32 |
| **Germany** | . | . | 12.53 | 12.68 | 12.84 | 12.99 | 13.14 | 13.3 | 13.45 | 13.61 |
| **Iceland** | . | . | . | . | 14.2 | 14.4 | 14.6 | 14.8 | 15 | 15.2 |
| **Italy** | . | . | . | 10.84 | 11.09 | 11.33 | 11.58 | 11.82 | 12.07 | 12.31 |
| **Netherlands** | . | . | . | 8.22 | 8.24 | 8.26 | 8.28 | 8.31 | 8.33 | 8.35 |
| **Norway** | 25.17 | 25.31 | 25.45 | 25.6 | 25.74 | 25.88 | 26.02 | 26.16 | 26.3 | 26.44 |
| **Spain** | . | . | . | 14.78 | 15.06 | 15.34 | 15.62 | 15.9 | 16.18 | 16.45 |
| **Sweden** | . | . | . | 9.93 | 10.01 | 10.09 | 10.17 | 10.25 | 10.33 | 10.41 |
| For each model. forecasts start after the last non-missing in the range of the requested estimation period. and end at the last period for which non-missing values of all the predictors are available or at the end date of the requested forecast period. whichever is earlier. | | | | | | | | | | |

***Table S8:*** *Assessment of reliability for ARIMA(0,1,0) models. Criteria include the assessment of fit metrics and the relative range of the confidential interval in 2030. Green coloured are characteristics that are considered with a good reliability, yellow-coloured with a moderate reliability and orange-coloured with a poor reliability.*

| **country** | **Croatia** | **Denmark** | **Estonia** | **Finland** | **Germany** | **Iceland** | **Italy** | **Netherlands** | **Norway** | **Spain** | **Sweden** |
| --- | --- | --- | --- | --- | --- | --- | --- | --- | --- | --- | --- |
| **fit metrics lithium forecast models** | - | moderate | poor | moderate | moderate | moderate | poor | moderate | good | poor | moderate |
| **relative range UCL and LCL lithium prediction** | - | 34.6% | 158.1% | 26.7% | 28.6% | 70.1% | 152.1% | 95.0% | 23.1% | 216.7% | 23.8% |
| **fit metrics antipsychotic**  **forecast models** | good | good | good | moderate | moderate | moderate | good | moderate | good | good | good |
| **range UCL and LCL**  **antipsychotics prediction** | 30.2% | 22.1% | 30.3% | 25.4% | 37.9% | 45.7% | 14.1% | 14.1% | 24.0% | 7.6% | 10.7% |
